# Persistent influence of the North Atlantic Oscillation on Late Holocene hydroclimate in southwestern Greenland

**DOI:** 10.1038/s41467-026-77084-0

**Published:** 2026-08-21

**Authors:** Johan C. Faust, Yang Zhang, Andreas Born, Dominik Hülse, Tilo von Dobeneck, Hartmut Schulz, Anjuly Janßen, Subhadip Mukherjee, Henrieka Detlef, Marit-Solveig Seidenkrantz, Rebecca Jackson, Jürgen Titschack, Anne de Vernal, Michal Kucera

**Affiliations:** 1 MARUM – Center for Marine Environmental Sciences, University of, Bremen, Germany; 2 BUKEA – Ministry for Environment, Climate, Energy and Agriculture, Hamburg, Germany; 3 Faculty of Geosciences, University of Bremen, Bremen, Germany; 4 Department of Earth Science, University of, Bergen, Norway; 5 Bjerknes Centre for Climate Research, Bergen, Norway; 6 Institut für Geowissenschaften, Eberhard-Karls-Universität, Tübingen, Germany; 7 Department of Geoscience, Aarhus University, Aarhus, Denmark; 8 Geotop, Université du Québec à, Montréal, QC Canada

**Keywords:** Palaeoclimate, Environmental impact, Climate-change impacts

## Abstract

Understanding how global climate forcing shapes regional hydroclimate on decadal to millennial timescales is critical for predicting the response of continental ice sheets to future warming. Because instrumental observations capture only a short interval, longer-term perspectives require proxy records. Here we present a high-resolution sedimentary record covering the entire Holocene from southwestern Greenland. We show that after the demise of local glaciers in the Early Holocene, the record allows evaluation of millennial to decadal variations in local precipitation. Comparison with North Atlantic Oscillation (NAO) reconstructions and climate model simulations demonstrates that positive NAO phases correspond to wetter but colder regional conditions. These findings suggest that centennial-scale patterns of ice mass balance during the late Holocene were influenced by precipitation variability, in addition to temperature changes. While the sensitivity to precipitation variability may change under a warmer climate, our results underscore the importance of integrating shifts in NAO-driven moisture delivery to accurately assess the future stability of the Greenland Ice Sheet.

## Abstract (web summary)

A 12,000-year marine sediment record from southwest Greenland identifies niobium as a hydroclimate proxy and reveals a persistent influence of the North Atlantic Oscillation on regional precipitation and glacier mass balance.

## Introduction

Arctic marine coastal areas with peripheral glaciers and ice caps are highly sensitive to global climate forcing due to Arctic amplification, the accelerated warming of the Arctic relative to the global mean^1^. With continued warming, meltwater from the Greenland Ice Sheet and Arctic glaciers is becoming an increasingly important source of contemporary sea-level rise^2^, and the channelling of the meltwater into the North Atlantic may affect large-scale oceanic circulation^3,4^. Thus, under a warming climate, understanding and predicting the interaction between the Arctic cryosphere and climate variability is of fundamental interest, especially given the far-reaching implications for marine ecosystems and global society. On millennial timescales, existing proxy records indicate that the long-term Arctic climate variability during the Holocene was driven by a gradual response to changes in summer insolation^5^. In contrast, the driving mechanisms of the subsequent centennial-scale variability remain contested, with numerous processes being invoked, such as solar irradiance, Atlantic meridional overturning circulation, and internal modes of atmospheric variability,e.g., refs. ^6–8^.

The western coast of southern Greenland is a strategic region to study the environmental response in relation to Greenland Ice Sheet (GIS) dynamics, changes in North Atlantic currents, and atmospheric circulation^9–11^. Modern observations reveal a clear relation between precipitation, temperature, and the large-scale atmospheric circulation pattern known as the North Atlantic Oscillation (NAO) in southern Greenland^10,12^. However, little is known about the historical behaviour of the NAO (before the pre-instrumental period), as NAO reconstructions are sparse, and only three records cover a time span beyond the past millennium^8,13,14^. Moreover, in coastal Greenland, challenges in obtaining high-resolution climate records that span the entire Holocene have hampered efforts to robustly determine the precise timing of climate change. Nearly all high-resolution records are from ice cores that document conditions over the GIS, far from the climate-sensitive southern Greenland coastal areas. Past fluctuations in glacier extent were commonly used to identify GIS response to Holocene climate variability and vice versa. These reconstructions are mainly constrained by lacustrine records and ^10^Be and radiocarbon-dated moraines^11,15–21^. However, based on these records, past glacial activity is difficult to reconstruct continuously and with high temporal resolution. Moreover, glacial response may react with a delay due to the inertia of ice caps. The linkage between NAO and climate variability on longer time scales and its role during abrupt climate change during the Holocene in (southern) Greenland is therefore still under debate ^e.g^^22,23^. For example, an antiphase centennial-scale variability between northwest Europe and southern Greenland climate has often been hypothesised but never shown beyond the late Holocene^17,23–25^.

**Fig. 1:**
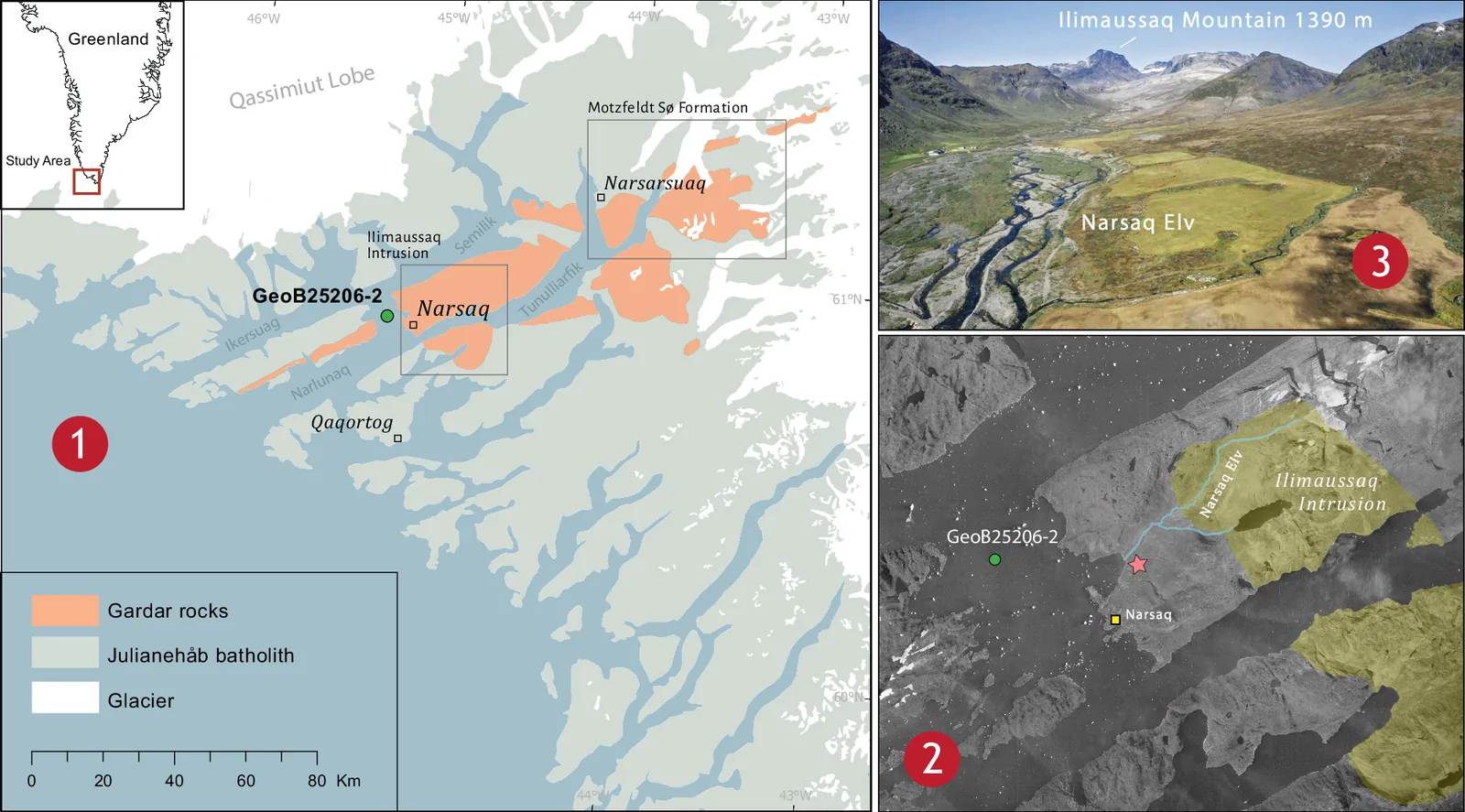
Regional geology and landscape setting of the Narsaq Sound core site. (1) Schematic geology and geomorphology map of the Narsarsuaq-Narsaq-Qaqortog region in southern Greenland (upper left inlet). The green dot marks the location of gravity core GeoB25206-2 in the Narsaq Sound close to the Narsaq settlement. The geological setting of the study area is characterised by two tectono-stratigraphic components comprising the older plutonic rocks of the Julianehab igneous complex (grey), formed during the Palaeoproterozoic Ketilidian orogen, and the younger Mesoproterozoic Gardar alkaline igneous province (orange), emplaced during episodes of continental rifting^29^. Parts of the Gardar intrusive complex are the IIímaussaq intrusion and Motzfeldt Sø formation (grey boxes)^30^. Concentrations of high field strength elements (HFSE) are exceptionally high in these units and considered as an indication of a major, yet undeveloped, niobium (Nb), tantalum (Ta), and rare earth element (REE) deposits^45,93^. (2) Aerial photography of the Narsaq region from 1985 ((CC BY 4.0) Agency for Climate Data). The Narsaq Elv river (blue line) originates in the central part of the Ilímaussaq Intrusion (yellow area) and discharges into the Narsaq Sound via a ~ 250 m wide delta. The red star marks the approximate location of the landscape photo (3) showing the Narsaq valley with the Ilimaussaq Mountain in the background (Copyright: Energy Transition Minerals).

During the past two decades, fjord sediments have been recognised as ideal archives for high-resolution reconstructions of past climate and environmental change ^e.g^^13,26,27^. In the present study, we focus on a sediment core from the Narsaq Sound (Fig. 1), a fjord system situated between the GIS and the Atlantic Ocean, ideally placed to identify past hydroclimate changes in relation to large-scale climate forcing factors. Specifically, we make use of the established XRF core scanning technique to test if the occurrence of unusually high concentrations of the element niobium (Nb) in distinct geological formations in the vicinity of Narsaq Sound can be used as a defined sediment source to track changes in river discharge during the past 12 ka.

## Environmental setting

The Narsarsuaq-Narsaq-Qaqortoq region is located on the west coast of Greenland, about 175 km from Kap Farvel, the southernmost point of Greenland. The area is characterised by a large system of more than 600 m deep fjords surrounded by mountains reaching 500 m near the coast and up to 1500 m further inland. These mountains are often covered by smaller ice caps. The GIS is generally less than 100 km away from the shoreline^28^, and several marine terminating ice-streams in the inner part of the fjords are substantial sources for icebergs^15^. The Narsaq Sound, located in the centre of the Narsarsuaq-Narsaq-Qaqortog region (Fig. 1), forms a 285 m deep, 5 km wide and about 12 km long connection between the Ikersuaq-Semilik and the Narlunaq-Tunulliarfik fjord systems. It is surrounded by up to ~1400 m high mountainous terrain. The main local sediment source is a larger meltwater river (Narsaq Elv) entering the Narsaq Sound through a ~ 250 m wide delta close to the town of Narsaq (Fig. 1). The river drains the small Narsaq Mountain Glacier (about 1.5 km^2^) in the area of the IIímaussaq intrusion, 10 km to the north-east of Narsaq. For a detailed description of the main geological and geographical attributes of the Narsarsuaq-Narsaq-Qaqortog region and the Narsaq Sound, we refer to Nørgaard-Pedersen and Mikkelsen^9^, Steenfelt, Kolb^29^, and Kokfelt, Weng^30^.

## Results

### Narsaq Sound deglaciated before surrounding land

**Fig. 2:**
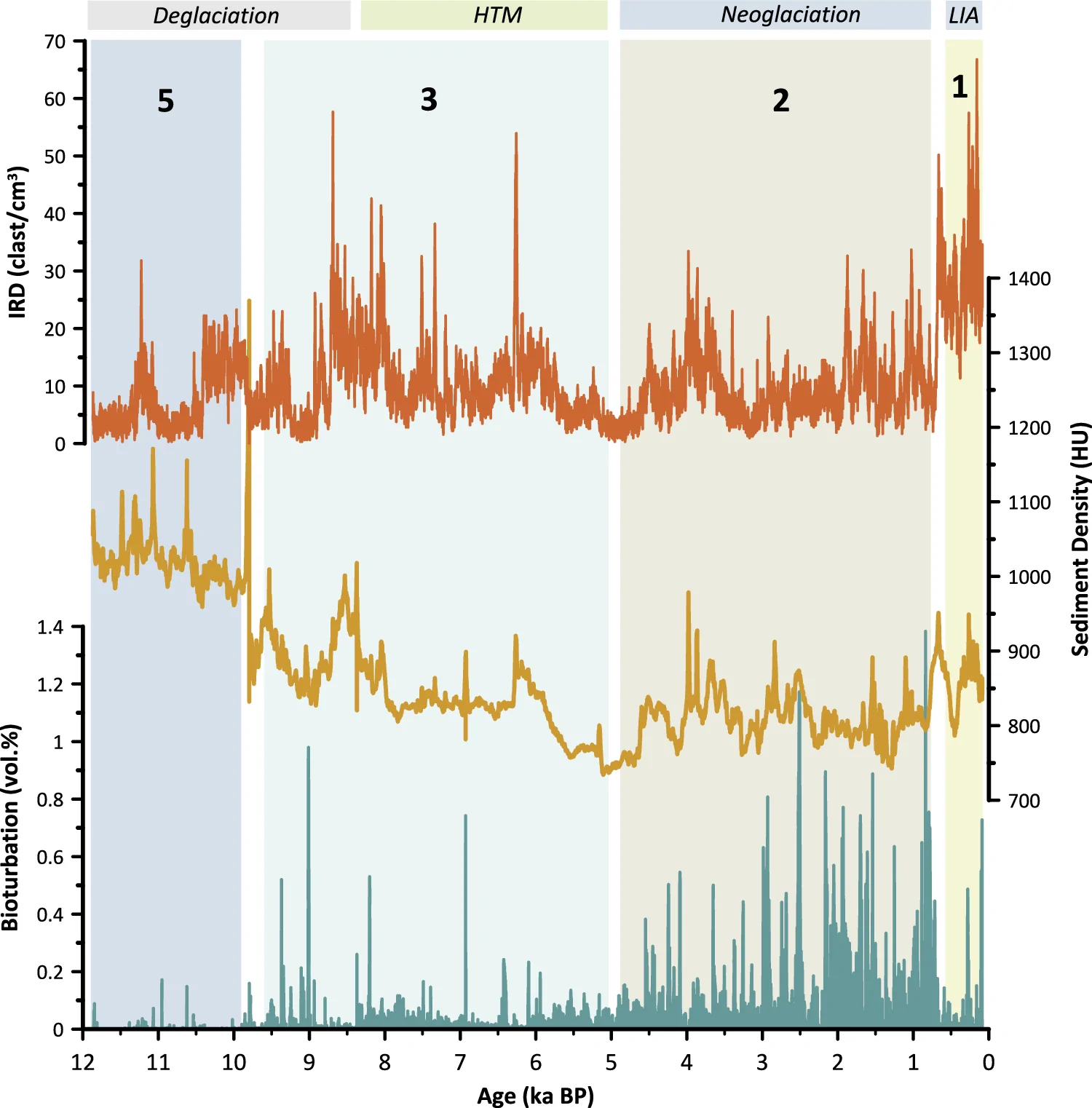
Sediment properties relate to Holocene climate evolution in Southwest Greenland. Sediment properties of gravity core GeoB25206-2 from the Narsaq Sound comprising bioturbation, apparent sediment density and IRD concentration, determined from high-resolution X-ray computed tomography. These parameters subdivide the sediment record into five lithological units shown by shaded areas. Unit four (not shown) represents a mass-wasting deposit occurring at ~ 9795 years BP (see Supplementary Fig. 5). The remaining units are related to the development of the climatic conditions in south Greenland from the early Holocene until the present: Deglaciation, Holocene Thermal Maximum (HTM), Neoglaciation and Little Ice Age (LIA).

During the Last Glacial Maximum and until about 15 ka BP (before present 1950), the GIS extended to the outer shelf, and all presently ice-free areas in southern Greenland were glaciated^31^. Rising temperatures at the Younger Dryas–Holocene transition (~11.6 ka BP) led to a fast retreat of the GIS from the outer coast towards its present extent with maximum recession rates of up to 100 m/yr^32,33^. However, the exact deglaciation timing of the Narsarsuaq-Narsaq-Qaqortog region is still under debate. Radiocarbon dates from basal lake sediments suggest that the GIS reached its present-day dimension (Fig. 1) between 10.6 and 9.5 ka BP^15,18^. A more recent study based on ^10^Be surface exposure ages indicates an earlier deglaciation of South Greenland at 11.1 ± 0.2 to 10.6 ± 0.5 ka BP^21^. The oldest radiocarbon age in our sediment core GeoB25206-2 at 1011 cm depth is ~11.3 ka BP (Supplementary Data 1). Large dark-grey patches, which appear before ~11.3 ka BP (Supplementary Fig. 1), are probably caused by iron-sulphide formation during sub- or anoxic bottom-water conditions. The interval (from 1011 cm down to the core base) is also marked by low amounts of Ice-Rafted Debris (IRD) and low signs of bioturbation (Fig. 2 and Supplementary Figs. 2, 3). We interpret these features as signs of restricted bottom-water exchange in the fjord system of the Narsarsuaq-Narsaq-Qaqortog region before the Younger Dryas–Holocene transition. The disappearance of the dark patches is accompanied by a distinct IRD layer at ~1000 cm, which suggests that the Narsaq Sound became at least periodically ice-free at about 11.3 ka BP, allowing the transport and melting of icebergs. The deglaciation time inferred from our sediment record aligns well with the estimated deglacial timings of the Bernstorff, Sermilik, and Kangerdlugssuaq fjord regions along the southeast margin of Greenland^34^. ^10^Be exposure dates indicate deglaciation of these areas between 11.8 and 10.4 ka BP and are proposed to coincide with the intrusion of warmer Atlantic water^34^. Indeed, water exchange between the Narsaq fjord system and the open ocean is relatively unrestricted, as there is no entrance sill; the fjord continues as a channel system 20–30 km out onto the shelf^9^. Since marine-terminating glaciers of the GIS margins can respond much faster to climate change^35^ than land-terminating margins^36,37^, we propose that the Narsaq fjord system deglaciated earlier than the surrounding land, probably due to the enhanced inflow of warmer Atlantic Ocean water at the end of the Younger Dryas^38,39^.

### Niobium as marker for hydroclimate change in southern Greenland

The element Niobium (Nb; formerly Columbium, Cb) is typically only present at very low concentrations in marine sediments and the continental crust (~11 ppm)^40,41^. Because of its economic importance in the electronics and steel industries, most environmental research on niobium has focused on geochemical mapping and resource exploration^40,42^. Beyond occasional use as a tracer of anthropogenic influences^41^, niobium has not been considered as a tracer of sediment and/or meltwater discharge in paleoenvironmental studies. Yet, niobium has no known biological function and behaves as a relatively immobile element in most geochemical environments^43^; in marine sediments, it is primarily supplied through terrigenous input derived from the weathering of continental rocks^44^. These properties make niobium a promising candidate as a marker of sediment provenance and past environmental change, particularly in settings where concentrations are elevated.

Greenland, known for its alkaline igneous provinces, has been the focus of rare earth element exploration for several decades. In particular, the alkaline complexes Motzfeldt Sø Formation and Ilímaussaq Intrusion close to Narsarsuaq and Narsaq (Fig. 1) host several world-class tantalum–niobium–REE deposits^45,46^. Accordingly, niobium concentrations in bedrock and stream sediments are exceptionally high in these areas, ranging between 0.14 and 1.0 wt.%^47,48^. Because of the Narsaq Sound bathymetry^9^, the delivery of lithic material to the location of our sediment core GeoB25206-2 is assumed to be dominated by sedimentary processes common to high latitude fjord settings, such as river transport fed by spring snow melting and runoff at ice sheet margins, as well as physical erosion of unglaciated coastal formations. Hence, we consider the changes in the elemental composition of the marine sediment core GeoB25206-2 to be highly constrained by the local response of terrigenous material transported into the fjord. Specifically, elevated niobium concentrations in marine sediments in the Narsarsuaq-Narsaq-Qaqortog region and in southern Greenland shelf sediments should indicate enhanced material input from the alkaline igneous provinces. Downcore variability in niobium should therefore reflect climatically induced environmental changes in the drainage basin, i.e., glacial melting, precipitation, and temperature changes.

**Fig. 3:**
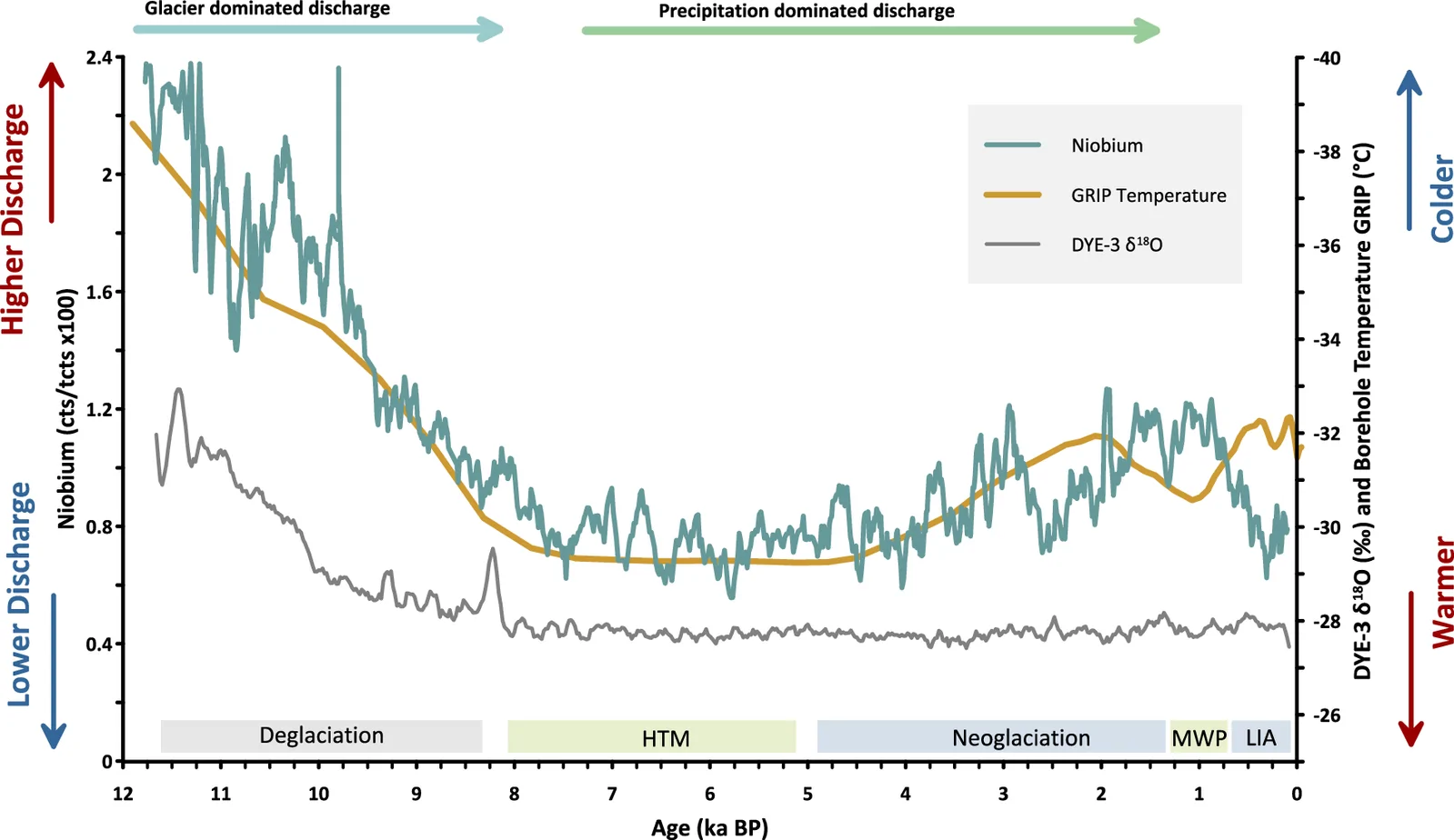
Niobium content in core GeoB25206-2 as marker for Holocene hydroclimate variability in Southwest Greenland. XRF core scanning result of Niobium from the Narsaq Sound (expressed as 5-pt. running average) compared to GRIP ice core borehole temperatures^52^ (yellow line) and δ^18^O from the DYE-3 ice core (grey line; 5-pt. running average)^53^ during the past 12 ka BP. Note: reversed y-axes of the ice core data. HTM Holocene Thermal Maximum, MWP Medieval Warm Period, LIA Little Ice Age.

In accordance with our newly inferred age of deglaciation in the Narsaq region, our record shows the highest niobium values during the Younger Dryas–Holocene transition (~11.6 ka BP) at the base of the core (Fig. 3). The niobium content gradually decreases until ~7.5 ka BP and stays relatively stable thereafter until about 4 ka BP. Values gradually increase again until about 1 ka BP and decrease towards the top of the core. An attribution of these changes to variable transport of niobium-rich sediments from the Ilímaussaq Intrusion mainly via the Narsaq Elv (Narsaq River; Fig. 1) is consistent with a scenario where the gradually decreasing niobium values during the early Holocene are related to the melting of local glaciers and subsequent enhanced freshwater inflow (Fig. 3). This assumption is supported by the reddish/pink sediment hue in the lower part of the core (Supplementary Fig. 1), which we attribute to the distinctive red-coloured Igaliko Sandstone, a common sedimentary rock formation of the Ilímaussaq Intrusion and the Gardar Province^49–51^. The proposed linkage between Greenland’s early Holocene deglaciation and our niobium record is further shown by the coherence between niobium content and borehole temperature and isotope (δ^18^O) measurements from GIS ice cores GRIP and DYE-3^52,53^ (Fig. 3). Both ice core records show an overall gradual warming trend until about 7.5 ka BP, resulting in a progressive decrease in meltwater discharge, due to the retreat and eventually demise of the local glaciers.

### Hydroclimate in southern Greenland driven by NAO variability

It is commonly proposed that during the Holocene Thermal Maximum (HTM, ~8–5 ka BP), elevated air temperatures^54,55^ and enhanced Atlantic water inflow^56–59^ have driven a major retreat of the southern GIS, which reached its minimum extent around ~4 ka BP, possibly tens of kilometres behind its present margin^17,60^. In relation to the shrinking GIS, tidewater glaciers are suggested to have become land-terminating during the HTM, causing a progressive reduction of IRD flux to the Narsarsuaq-Narsaq-Qaqortog fjord system from 8 to 4.8 ka BP^9^. Consistent with the proposed GIS retreat, our IRD, sediment density and magnetic susceptibility (MS) records also show a gradual decrease from ~8.7 to 4.9 ka BP (Fig. 2 and Supplementary Fig. 5). However, our high-resolution IRD record also reveals periods of enhanced IRD fluxes to our coring site, indicating increased iceberg calving probably due to glacier surges. In addition, the near-continuous presence of IRD during the HTM indicates that marine-terminating ice streams must have persisted in the Narsarsuaq-Narsaq-Qaqortoq region. Since sedimentation rates remain relatively high and stable during this period (Supplementary Fig. 6), the decrease in sediment density and magnetic susceptibility may be related to an increased transport of fine-grained material sourced, e.g. from meltwater tributaries. The locally sourced niobium content shows small-scale variability, but no gradual change concurrent with the ice core data (Fig. 3).

Due to decreasing isolation, northern-hemisphere temperatures started to decline after the HTM at about 5 ka BP. The gradual cooling associated with the initiation of the Neoglacial Period lasted until ~1150 years BP ^e.g^ ^68^ and was followed by the Medieval Warm Period (MWP) and the Little Ice Age (LIA; Fig. 3). In accordance with late Holocene climate change, our niobium record shows a gradual increase from ~5 ka BP until ~1 ka BP, interrupted by a short interval of lower values at ~2.5 ka BP. During the later part of the Neoglaciation, niobium shows a relatively steep decrease reaching its lowest values during the LIA at ~250 years BP (Fig. 3). The comparison with ice core borehole temperatures shows a good agreement between the niobium record and GIS temperatures until ~2 ka BP. However, a response to the temperature rise during the Medieval Warm Period is not observed in the niobium record.

**Fig. 4:**
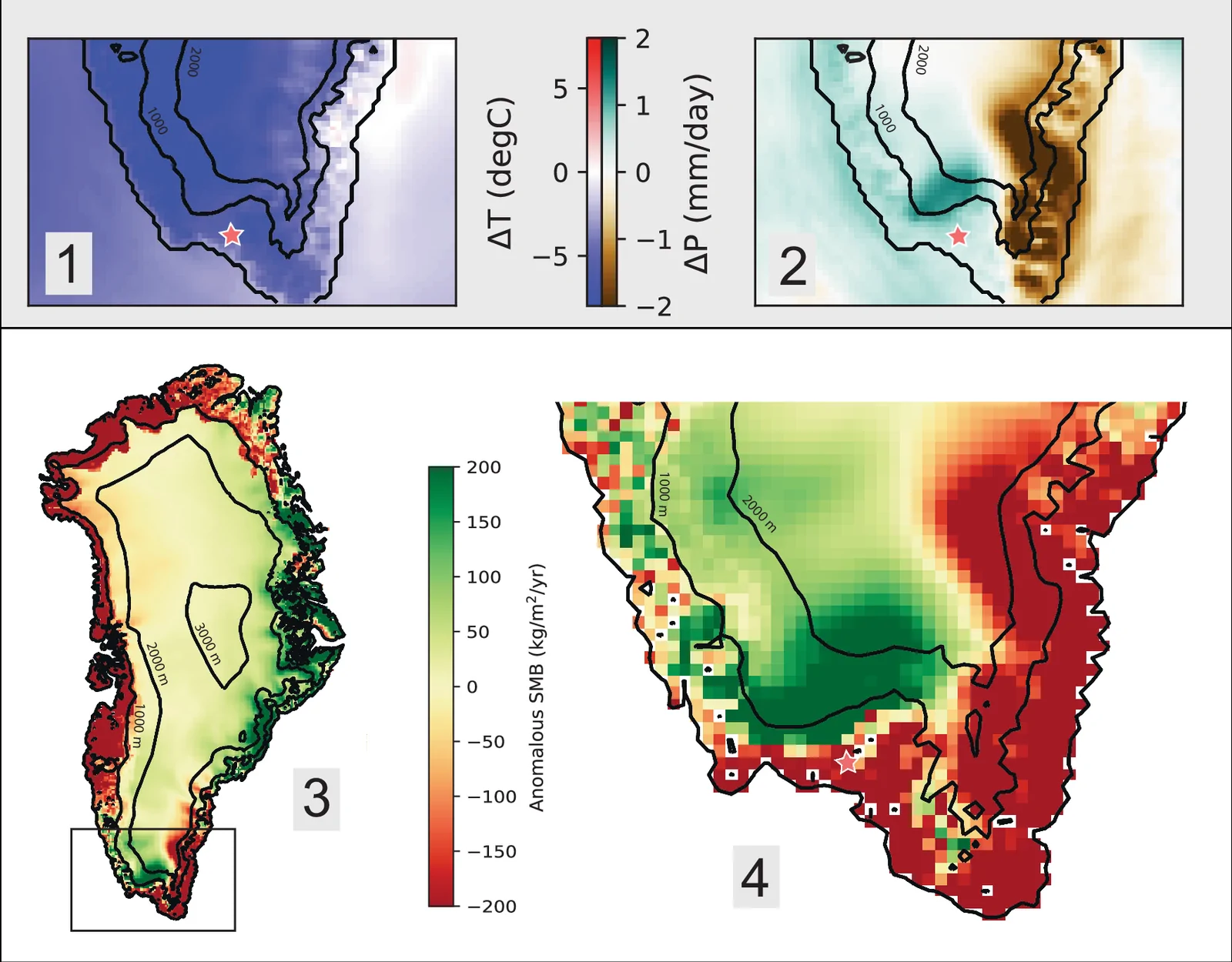
NAO-driven anomalies in temperature, precipitation, and surface mass balance. The regional imprint of the NAO climatic anomaly on the temperature (1) and precipitation (2) in southern Greenland. The effect of the NAO is expressed as the difference between the NAO+ and the NAO- state extracted from the Modèle Atmosphérique Régional (MAR) v3.14^87^. Surface mass balance (SMB) anomaly (NAO+ minus NAO-) is shown for all of Greenland (3) and southern Greenland (4). The black rectangle in panel 3 indicates the section shown in panels 1, 2, and 4. The red star marks the position of the sediment core GeoB25206-2 from the Narsaq Sound. Simulations were run for 100 years; NAO index data were provided by the Climate Analysis Section, NCAR, Boulder, USA^94^.

Given that we argue that the niobium content in Narsaq Sound sediments is related to river discharge and therefore precipitation and snow melting, it might seem contradictory that higher temperatures during the HTM and lower temperatures during the Neoglaciation are related to lower and higher fluxes of niobium via river discharge, respectively. We find that this observation can be explained by the dominant mode of atmospheric circulation in the North Atlantic region, the NAO^61^. The NAO is defined by the atmospheric pressure difference between the Icelandic Low and the Azores High and is most pronounced during winter. In northwestern Europe and in southwest Greenland, positive NAO (NAO + ) relates to periods of higher precipitation and vice versa (Fig. 4 and Supplementary Fig. 7)^61^. However, in contrast to northwestern Europe, air temperature in southwest Greenland is negatively correlated with the NAO^8,10,12,62^. Hence, in southwestern Greenland, NAO+ (NAO-) relates to lower (higher) temperatures and higher (lower) precipitation and river discharge.

**Fig. 5:**
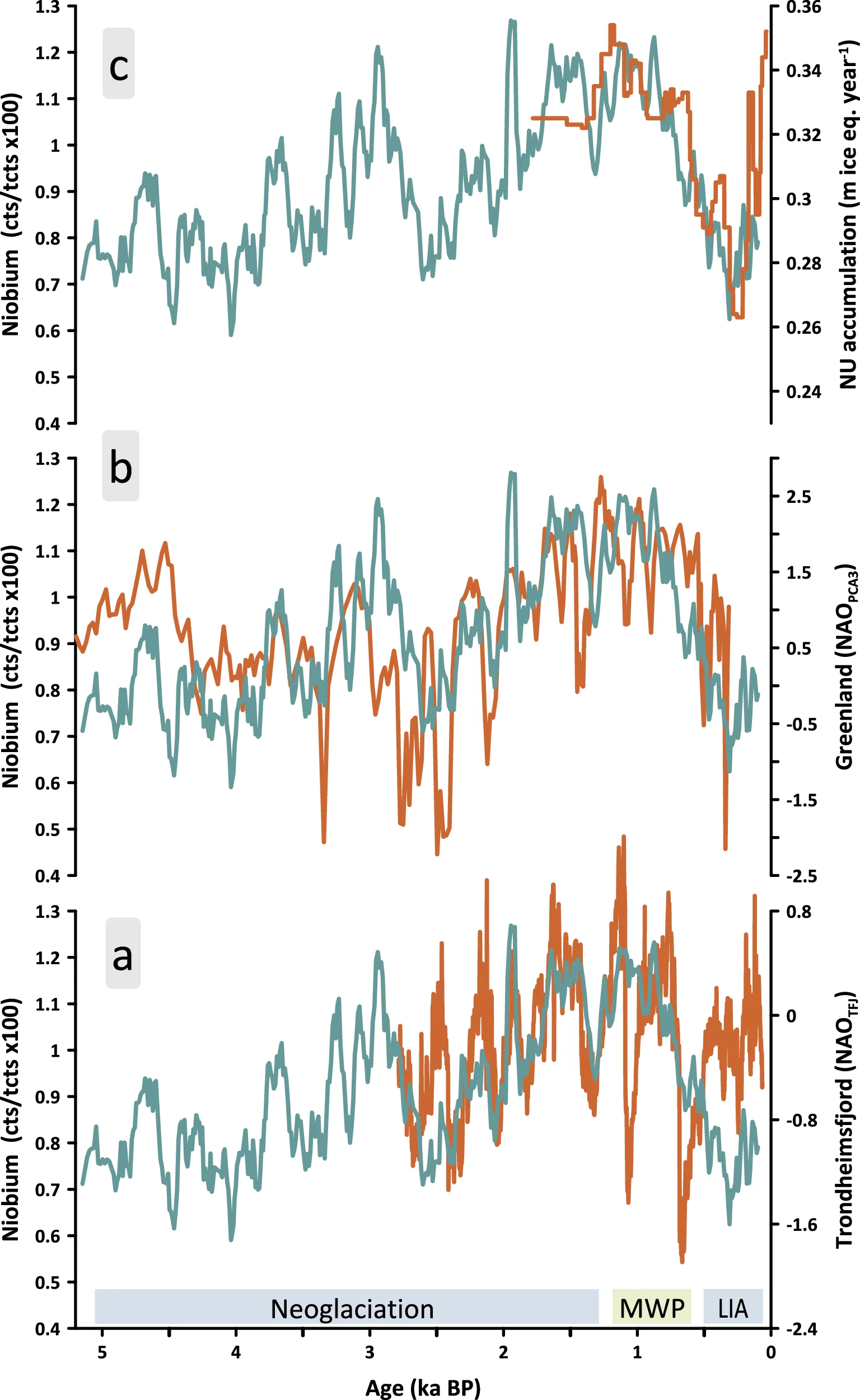
Niobium record compared with North Atlantic Oscillation reconstructions and regional ice accumulation in Greenland. Comparison of the GeoB25206-2 Niobium record (green line; 5-pt. running av.) with North Atlantic Oscillation (NAO) reconstructions based on **a** marine sediments from the Trondheimsfjord in mid-Norway (NAO_TJF_)^13^ and **b** lake sediments from west-Greenland (NAO_PCA3_)^8^. A comparison between Niobium and Nuussuaq (NU) ice cap accumulation history^70^ in west-Greenland is shown in (**c**).

In accordance with our niobium record, NAO reconstructions from lake sediments in southwestern Greenland and from Norwegian fjord sediments reveal a gradual increase and a pronounced positive NAO phase at the end of the Neoglaciation with highest values during the Medieval Warm Period, ~1 ka ago^8,13^ (Fig. 5a, b). Smoothing- and moving-window correlation analyses of our Nb record with the Greenland and Trondheimsfjord NAO indices reveal consistently positive Pearson correlation coefficients (Tab. 1 and Supplementary Figs. 8, 9), reflecting the linkage of atmospheric circulation and Nb content in Narsaq Sound sediments. Correlation coefficients (r) increase systematically as the smoothing window widens and strengthen and stabilise with increasing moving window size. This indicates that the coupling between the proxies is primarily expressed on centennial to millennial timescales, while their high-frequency variability is dominated by noise and local-to-regional environmental and climatic processes. The coherence of the Nb record with the NAO indices from Greenland and Norway (Fig. 5, Tab. 1 and Supplementary Figs. 8, 9) suggests a scenario where, in the absence of large local glaciers, freshwater and thus terrigenous material discharge through the Narsaq Elv river into the Narsaq Sound followed the NAO-driven increase in precipitation until the end of the Neoglaciation.

Our results indicate that NAO variability influenced regional precipitation, which in turn affected local ice-sheet surface mass balance (SMB). While our proxy record does not directly reconstruct glacier or ice-sheet dynamics and therefore cannot, on its own, demonstrate that NAO variability caused measurable changes in glacier behaviour, it also does not exclude the possibility of such changes. Instead, when considered together with previous observational and modelling studies, e.g., refs. ^63–67^, our findings support the interpretation that persistent NAO-driven hydroclimate variability likely influenced glacier mass balance and, consequently, glacier behaviour in southern Greenland. So far, late Holocene glacial activity in southern Greenland is complex and not fully understood. Depending on location and altitude, different responses of the GIS and local glaciers to abrupt climate change during the transition between warmer and colder periods of the Roman Warm Period, Dark Ages, Medieval Warm Period and Little Ice Age have been reported^18,20,68,69^. At the onset of the Little Ice Age (~750 years BP), the IRD content in our core shows a sudden rise accompanied by a decrease in bioturbation and a reduction in the niobium content (Figs. 2, 3). An increase in sedimentary IRD content is often interpreted as a sign of enhanced iceberg calving, faster iceberg melting, a change in the direction of transport, higher sediment load, or a combination of these. Although we consider a change in transport direction negligible in the small fjord system, a change in sediment load remains possible. Nevertheless, in line with a previous IRD study from the Narsaq Sound^9^, we suggest that the very high IRD content over the past 800 years shows increased iceberg calving from marine-terminating glaciers in the Narsarsuaq-Narsaq-Qaqortoq region. Moreover, the decreasing niobium content points to a pronounced regional hydroclimate shift. This interpretation is supported by a strong coherence between our niobium record and recent findings from a coastal West Greenland ice core, showing a 20% reduction in average snow accumulation from the Medieval Warm Period to the Little Ice Age, followed by a > 40% increase since the early 18th century (Fig. 5c)^70^. The coherence between the two records is further confirmed by our correlation analysis, which reveals the strongest and most persistent relationship between the Nb record and Nuussuaq ice cap accumulation history (Table 1 and Supplementary Figs. 8, 9)^70^, supporting our interpretation of Nb as a hydroclimate proxy. Moreover, investigations of lacustrine biomarker data from a lake located 35 km northeast of our coring site revealed a prolonged drying trend which peaked in the 16th century during the LIA^71^. Consistent with our interpretation, both studies attribute the regional hydroclimate changes to a long-term shift from predominantly positive NAO conditions during the MCA to predominantly negative NAO conditions during the LIA.

**Table 1:** Correlation coefficients (r) in function of smoothing window size for the datasets shown in Fig. 5, computed over their respective overlapping intervals with the Niobium record

Window size (years)	NU accumulation^73^	Greenland^8^	Trondheimsfjord^13^
50	0.73	0.42	0.43
75	0.76	0.44	0.44
100	0.79	0.46	0.46
125	0.81	0.47	0.47
150	0.85	0.50	0.50
175	0.87	0.51	0.52
200	0.88	0.52	0.53
225	0.90	0.53	0.56
250	0.92	0.54	0.61

Correlations were evaluated over the full time series (i.e., without a moving-window approach) after interpolation to a common time grid (dt = 20 years). For additional details and sensitivity analyses, see Supplementary Data 4.

We note that, in addition to changes in the NAO, other factors likely affected the sedimentary record and hydroclimate during the Holocene. For example, sea surface temperature (SST) variability may have influenced regional hydroclimate and could have contributed to the observed trends. However, continuous, high-resolution SST reconstructions from coastal southern Greenland, covering the full Holocene and needed to test such associations, are currently lacking. Moreover, changes in the Atlantic meridional overturning circulation (AMOC) are unlikely to have been a primary driver of the observed hydroclimatic changes in the time period of interest, as there is currently no evidence for sufficient changes in the AMOC during the past 5 ka, e.g., ref. ^72^.

Collectively, our results indicate that NAO variability appears to have persistently modulated southern Greenland hydroclimate, and changes in hydroclimate likely influenced local glacier behaviour and mass balances. The LIA was characterised by drier conditions and enhanced glacier instability, in antiphase with central Greenland temperature trends and out of phase with northwestern Europe. This highlights the spatial complexity of Greenland’s climate system and the non-linear response of the GIS to atmospheric forcing. Importantly, while ongoing anthropogenic warming is accelerating GIS mass loss and global sea-level rise, some model projections suggest that increasing CO₂ concentrations may favour a more positive and less variable NAO state^73,74^. Surface mass balance (SMB) calculations under NAO+ conditions (Fig. 4) are consistent with the hydroclimatic patterns inferred from our late Holocene sedimentary record. This suggests that the southwestern GIS margins may be more stable in the future, with reduced glacier retreat compared to other areas of Greenland. However, future analyses using regional climate and ice-sheet model projections will be needed to determine whether enhanced precipitation could locally offset increased ablation and potentially promote localised glacier growth, or whether continued atmospheric warming remains the dominant control on glacier mass balance. Moreover, the sharp regional contrasts of the SMBs underline the need to consider regional climate patterns when extrapolating from local glacier to whole-ice-sheet behaviour.

## Methods

### Narsaq Sound sediment core description and magnetic susceptibility

Sediment core GeoB25206-2 was collected during the MSM111 expedition onboard the German research vessel Maria S. Merian in September 2022^75^ from Narsaq Sound in South Greenland (Fig. 1; 60°56.200´N, 46°9.300´W; water depth 285 m). The coring location was chosen based on bathymetry data from Nørgaard-Pedersen and Mikkelsen^9^. The 10.83 m long and 12 cm wide gravity core was cut into one-metre sections. Their magnetic susceptibility (MS) was measured on board in 1 cm steps with the MARUM multi-sensor core logging system equipped with a Bartington Ltd. MS2C loop sensor^75^. Visual core description and high-quality optical imaging, using the smartCIS 1600 Line Scanner at MARUM, Bremen, were conducted during the MSM111 sampling party in early 2023.

### Chronology

The Chronology of core GeoB25206-2 is based on seven AMS radiocarbon (^14^C) dates from mixed benthic foraminifera assemblages using the Mini Carbon Dating System (MICADAS) at the Alfred Wegener Institute in Bremerhaven, Germany (Supplementary Data 1)^76^. As the MS record of the nearby sediment core Ga3-2^9^ and the GeoB25206-2 core revealed a high degree of covariation (Supplementary Fig. 4), three anchor points (Supplementary Figs. 4, 5) allowed us to transfer eight AMS ^14^C dates of core Ga3-2 to the age-depth model of GeoB25206-2 (Supplementary Fig. 6). The age-depth model was established by using the BACON age modelling package in R^77^, applying MARINE20 and a reservoir correction (ΔR) of -74 ± 24 years^78^.

### X-ray fluorescence

X-ray fluorescence (XRF) core scanning was accomplished on the archive halves at 1 cm intervals using the AVAATECH XRF Core Scanner (slit size 10 mm with 10 kV and 30 kV) at MARUM, Bremen. The split core surface was covered with a 4 µm thin SPEX Certi Prep Ultralene foil. The iterative least squares software package Win AXIL by Canberra Eurisys was used to convert the raw X-Ray spectra to individual elemental counts. To minimise bias by varying water content and sediment properties, XRF results were normalised to the sum of all counts per sample^79,80^ and are accordingly reported as counts/total counts (cts/tcts).

### Computed tomography

A Philips Brilliance iCT Elite 256 computed tomograph (CT) scanner at the hospital Klinikum Bremen-Mitte, Germany, was used to generate CT image stacks with a X-ray source voltage of 120 kV and a current of 300 mA. The system has a resolution of 0.293 mm in the x and y directions and of 0.625 mm in the z direction (collimation; 0.3 mm reconstruction interval). Images were reconstructed using the filtered Back Projection (fBP) mode and a bone kernel (YB (Enhanced)). CT data were processed using the ZIB edition of the Amira software (version 2021.08)^81^ following the methodology described by Bartels, Titschack^82^. Core liners were removed from all scans and subsequently merged into one dataset. High-density constituents as well as open bioturbation (low density) were segmented with the marker-based watershed algorithm. Markers were set by thresholding. High-density constituents >~1 mm consist of spherical (lithic) clasts and tubular lithified bioturbation traces. These two constituents were separated by using object shape parameters: minimum length >2 mm and ratio of maximum inner radius/maximum diameter <0.5^83^. Additionally, the X-ray attenuation of the matrix sediment, measured in Hounsfield unit (HU),—a proxy for wet bulk density—was determined with the Material Statistics module (statistics per slice and label) after reducing the matrix sediment label by two voxels to exclude potential marginal effects. Data were subsequently grouped into 10 mm bins with the NumPy^84^ and pandas (The Pandas Development Team 2020) cut and GroupBy-methods.

### Numerical modelling

We performed two simulations, NAO+ and NAO-, using the snow surface mass and energy balance model BESSI^85,86^. Both simulations were run for 100 years with atmospheric forcing from the regional climate model MAR^87^ to allow the snow layer to reach equilibrium. The NAO+ simulation used ten years with an NAO index at least one standard deviation above the mean (1992, 1993, 1995, 1999, 2000, 2005, 2008, 2012, 2015, 2020). The NAO- simulation used ten years with at least one standard deviation below the mean (1966, 1969, 1970, 1977, 1978, 1979, 1985, 1996, 2010, 2011). These ten-year sequences were repeated ten times to produce the 100-year simulations. NAO index data were provided by the Climate Analysis Section, NCAR, Boulder, USA^61^.

## Supplementary information

Supplementary Information

Description of Additional Supplementary File

Supplementary Data 1

Supplementary Data 2

Supplementary Data 3

Supplementary Data 4

Transparent Peer Review file

## Data Availability

The X-ray fluorescence (XRF; doi.pangaea.de/10.1594/PANGAEA.983542)^88^, CT 10.1594/PANGAEA.982944^89^, electrical conductivity, porosity and density 10.1594/PANGAEA.984144^90^, magnetic susceptibility 10.1594/PANGAEA.983992^91^, visual core description and line scanner images (doi.pangaea.de/10.1594/PANGAEA.983000)^92^ data generated in this study have been deposited in the data repository PANGAEA. The source data for figures in the text are available within the Supplementary Data 1-4.

## References

[1] Yamanouchi T., Takata K. Rapid change of the Arctic climate system and its global influences - Overview of GRENE Arctic climate change research project (2011–2016). *Polar Sci.* **25**, 100548 (2020).

[2] Stokes, C. R., Bamber, J. L., Dutton, A. & DeConto, R. M. Warming of +1.5 degrees C is too high for polar ice sheets. *Commun. Earth Environ.* **6**, 351 (2025). 40406387 10.1038/s43247-025-02299-w PMC12092291

[3] Böning, C. W., Behrens, E., Biastoch, A., Getzlaff, K. & Bamber, J. L. Emerging impact of Greenland meltwater on deepwater formation in the North Atlantic Ocean. *Nat. Geosci.* **9**, 523–527 (2016).

[4] Martin, T. & Biastoch, A. On the ocean’s response to enhanced Greenland runoff in model experiments: relevance of mesoscale dynamics and atmospheric coupling. *Ocean Sci.* **19**, 141–167 (2023).

[5] Wanner, H., Solomina, O., Grosjean, M., Ritz, S. P. & Jetel, M. Structure and origin of Holocene cold events. *Quat. Sci. Rev.* **30**, 3109–3123 (2011).

[6] Mehling O., Bellomo K., von Hardenberg J. Centennial-scale variability of the Atlantic meridional overturning circulation in CMIP6 models shaped by Arctic–North Atlantic interactions and sea ice biases. *Geophys. Res. Lett.* **51**, 110791 (2024).

[7] Moffa-Sánchez, P., Born, A., Hall, I. R., Thornalley, D. J. R. & Barker, S. Solar forcing of North Atlantic surface temperature and salinity over the past millennium. *Nat. Geosci.* **7**, 275–278 (2014).

[8] Olsen, J., Anderson, N. J. & Knudsen, M. F. Variability of the North Atlantic Oscillation over the past 5200 years. *Nat. Geosci.* **5**, 808–812 (2012).

[9] Nørgaard-Pedersen, N. & Mikkelsen, N. 8000 year marine record of climate variability and fjord dynamics from Southern Greenland. *Mar. Geol.* **264**, 177–189 (2009).

[10] Hanna E., Cappelen J. Recent cooling in coastal southern Greenland and relation with the North Atlantic Oscillation. *Geophys. Res. Lett.* **30**, 015797 (2003).

[11] Larsen, N. K. et al. Holocene ice marginal fluctuations of the Qassimiut lobe in South Greenland. *Sci. Rep.* **6**, 22362 (2016). 26940998 10.1038/srep22362 PMC4778018

[12] Bromwich, D. H., Chen, Q. -s, Li, Y. & Cullather, R. I. Precipitation over Greenland and its relation to the North Atlantic Oscillation. *J. Geophys. Res. Atmos.* **104**, 22103–22115 (1999).

[13] Faust, J. C., Fabian, K., Milzer, G., Giraudeau, J. & Knies, J. Norwegian fjord sediments reveal NAO related winter temperature and precipitation changes of the past 2800 years. *Earth Planet. Sci. Lett.* **435**, 84–93 (2016).

[14] Trouet, V. et al. Persistent positive North Atlantic Oscillation mode dominated the medieval climate Anomaly. *Science* **324**, 78–80 (2009). 19342585 10.1126/science.1166349

[15] Weidick, A., Kelly, M. & Bennike, O. Late Quaternary development of the southern sector of the Greenland Ice Sheet, with particular reference to the Qassimiut lobe. *Boreas* **33**, 284–299 (2004).

[16] Levy, L. B. et al. Multi-phased deglaciation of south and southeast Greenland controlled by climate and topographic setting. *Quaternary Sci. Rev.* **242**, 106454 (2020).

[17] Larsen, N. K. et al. The response of the southern Greenland ice sheet to the Holocene thermal maximum. *Geology* **43**, 291–294 (2015).

[18] Larsen, N. K. et al. Restricted impact of Holocene climate variations on the southern Greenland Ice Sheet. *Quat. Sci. Rev.* **30**, 3171–3180 (2011).

[19] Larocca L. J., Axford Y., Bjørk A. A., Lasher G. E., Brooks J. P. Local glaciers record delayed peak Holocene warmth in south Greenland. *Quaternary Sci. Rev.* **241**, 106421 (2020).

[20] Puleo, P. J. K. & Axford, Y. Duration and ice thickness of a Late Holocene outlet glacier advance near Narsarsuaq, southern Greenland. *Climate* **19**, 1777–1791 (2023).

[21] Carlson, A. E. et al. Earliest Holocene south Greenland ice sheet retreat within its late Holocene extent. *Geophys. Res. Lett.* **41**, 5514–5521 (2014).

[22] Lasher, G. E. & Axford, Y. Medieval warmth confirmed at the Norse Eastern Settlement in Greenland. *Geology* **47**, 267–270 (2019).

[23] Andresen, C. S., Björck, S., Bennike, O. & Bond, G. Holocene climate changes in southern Greenland: evidence from lake sediments. *J. Quat. Sci.* **19**, 783–795 (2004).

[24] Seidenkrantz, M.-S. et al. Variable North Atlantic climate seesaw patterns documented by a late Holocene marine record from Disko Bugt, West Greenland. *Mar. Micropaleontol.* **68**, 66–83 (2008).

[25] Seidenkrantz, M. S. et al. Hydrography and climate of the last 4400 years in a SW Greenland Fjord: implications for Labrador Sea palaeoceanography. *Holocene* **17**, 387–401 (2007).

[26] Howe, J. A. et al. Fjord systems and archives: a review. *Geol. Soc. Lond. Spec. Publ.* **344**, 5–15 (2010).

[27] Bianchi, T. S. et al. Fjords as Aquatic Critical Zones (ACZs). *Earth-Sci. Rev.* **203**, 103145 (2020).

[28] Weidick, A., Bøggild, C. E. & Knudsen, N. T. Glacier inventory and atlas of West Greenland. *Rapp. Grønlands Geologiske Undersøgelse* **158**, 1–194 (1992).

[29] Steenfelt, A., Kolb, J. & Thrane, K. Metallogeny of South Greenland: a review of geological evolution, mineral occurrences and geochemical exploration data. *Ore Geol. Rev.* **77**, 194–245 (2016).

[30] Kokfelt T., Weng W., Willerslev E. Geological map of South and South West Greenland-1: 100,000. (Geological Survey of Denmark and Greenland, 2019).

[31] Funder S., Kjeldsen K. K., Kjær K. H., Ó Cofaigh C. The Greenland ice sheet during the past 300,000 years: a review. *Quaternary Glaciations - Extent and Chronology - A Closer Look* **15**, 699–713 (2011).

[32] Lesnek A. J., Briner J. P., Young N. E., Cuzzone J. K. Maximum Southwest Greenland ice sheet recession in the Early Holocene. *Geophys. Res. Lett.* **47**, 083164 (2020).

[33] Taylor, K. C. et al. The Holocene Younger Dryas transition recorded at Summit, Greenland. *Science* **278**, 825–827 (1997).

[34] Dyke, L. M. et al. Evidence for the asynchronous retreat of large outlet glaciers in southeast Greenland at the end of the last glaciation. *Quat. Sci. Rev.* **99**, 244–259 (2014).

[35] Nick, F. M. et al. Future sea-level rise from Greenland’s main outlet glaciers in a warming climate. *Nature* **497**, 235–238 (2013). 23657350 10.1038/nature12068

[36] Kelley, S. E., Briner, J. P., Young, N. E., Babonis, G. S. & Csatho, B. Maximum late Holocene extent of the western Greenland Ice Sheet during the late 20th century. *Quat. Sci. Rev.* **56**, 89–98 (2012).

[37] Tarasov, L. & Richard Peltier, W. Greenland glacial history and local geodynamic consequences. *Geophys. J. Int.* **150**, 198–229 (2002).

[38] Rainsley, E. et al. Greenland ice mass loss during the Younger Dryas driven by Atlantic meridional overturning circulation feedbacks. *Sci. Rep.* **8**, 11307 (2018). 30093676 10.1038/s41598-018-29226-8 PMC6085367

[39] Menviel, L., Timmermann, A., Timm, O. E. & Mouchet, A. Deconstructing the Last Glacial termination: the role of millennial and orbital-scale forcings. *Quat. Sci. Rev.* **30**, 1155–1172 (2011).

[40] Liu, H. et al. Continental-scale distribution of niobium and tantalum in catchment sediments throughout China: prospecting implications from the China geochemical Baselines project. *Ore Geol. Rev.* **150**, 105189 (2022).

[41] Sutliff-Johansson, S. et al. Tracing anthropogenic sources of Tantalum and Niobium in Bothnian Bay sediments, Sweden. *J. Soils Sediment.* **21**, 1488–1503 (2020).

[42] Simandl, G. J. et al. Applicability of handheld X-Ray fluorescence spectrometry in the exploration and development of carbonatite-related niobium deposits: a case study of the Aley Carbonatite, British Columbia, Canada. *Geochem. Exploration Environ. Anal.* **14**, 211–221 (2014).

[43] Åström, M. E., Peltola, P., Virtasalo, J. J., Kotilainen, A. T. & Salminen R. Niobium in boreal stream waters and brackish-water sediments. *Geochem.-Explor Env A* **8**, 139–148 (2008).

[44] Firdaus, M. L., Mashio, A. S., Obata, H., McAlister, J. A. & Orians, K. J. Distribution of zirconium, hafnium, niobium and tantalum in the North Atlantic Ocean, northeastern Indian Ocean and its adjacent seas. *Deep Sea Res. Part I: Oceanogr. Res. Pap.* **140**, 128–135 (2018).

[45] Tukiainen T. The Motzfeldt of the Igaliko Nepheline Syenite Complex, South Greenland - A major resource of REE elements. *1st European Rare Earth Resources Conference* Milos (GEUS, 2014).

[46] Schønwandt H. K., Barnes G. B., Ulrich T. A description of the world-class rare earth element deposit, Tanbreez, South Greenland. *Rare Earths Industry* **10**, pp 73–85 (2016).

[47] Williams-Jones, A. E. & Vasyukova, O. V. Niobium, Critical Metal, and Progeny of the Mantle. *Econ. Geol.* **118**, 837–855 (2023).

[48] Steenfelt A., Olsen S., Heijboer T. Geochemical analyses of Stream sediment samples from Greenland. V2 ed: (GEUS Dataverse, 2023).

[49] Poulsen, V. The sandstones of the Precambrian Eriksfjord formation in South Greenland. *Rapp. Gr.ønlands Geologiske Undersøgelse* **2**, 1–16 (1964).

[50] Scharbert, H. G. A sandstone dyke in the Julianehåb granite of Qeqertarssuaq, Julianehåb district. *Med. Dan. Geologisk Foren.* **15**, 183–188 (1963).

[51] Matthews, D. Geological History of Greenland: Four Billion Years of Earth Evolution. Niels Henriksen. 2008. Copenhagen: Geological Survey of Denmark and Greenland (GEUS). 272 p, illustrated, hard back. ISBN 978-87-7871-211-0.£ 44. *Polar Rec.* **46**, 90–90 (2010).

[52] Dahl-Jensen, D. et al. Past temperatures directly from the Greenland ice sheet. *Science* **282**, 268–271 (1998). 9765146 10.1126/science.282.5387.268

[53] Vinther, B. M. et al. Holocene thinning of the Greenland ice sheet. *Nature* **461**, 385–388 (2009). 19759618 10.1038/nature08355

[54] Fréchette, B. & de Vernal, A. Relationship between Holocene climate variations over southern Greenland and eastern Baffin Island and synoptic circulation pattern. *Climate* **5**, 347–359 (2009).

[55] Wooller, M. J. et al. Quantitative paleotemperature estimates from d18O of chironomid head capsules preserved in arctic lake sediments. *J. Paleolimnol.* **31**, 267–274 (2004).

[56] Jennings, A., Andrews, J. & Wilson, L. Holocene environmental evolution of the SE Greenland Shelf North and South of the Denmark Strait: Irminger and East Greenland current interactions. *Quat. Sci. Rev.* **30**, 980–998 (2011).

[57] Jennings, A. E. et al. Paleoenvironments during Younger Dryas-Early Holocene retreat of the Greenland Ice Sheet from outer Disko Trough, central west Greenland. *J. Quat. Sci.* **29**, 27–40 (2013).

[58] Telesiński, M. M., Spielhagen, R. F. & Lind, E. M. A high-resolution Lateglacial and Holocene palaeoceanographic record from the Greenland Sea. *Boreas* **43**, 273–285 (2013).

[59] Lloyd, J. M., Kuijpers, A., Long, A., Moros, M. & Park, L. A. Foraminiferal reconstruction of mid- to late-Holocene ocean circulation and climate variability in Disko Bugt, West Greenland. *Holocene* **17**, 1079–1091 (2007).

[60] Lecavalier, B. S. et al. A model of Greenland ice sheet deglaciation constrained by observations of relative sea level and ice extent. *Quat. Sci. Rev.* **102**, 54–84 (2014).

[61] Hurrell, J. W. Decadal trends in the North Atlantic Oscillation: regional temperatures and precipitation. *Science* **269**, 676–679 (1995). 17758812 10.1126/science.269.5224.676

[62] Wanner, H. et al. North atlantic oscillation – concepts and studies. *Surv. Geophys.* **22**, 321–381 (2001).

[63] Calder C. A., Craigmile P. F., Mosley-Thompson E. Spatial variation in the influence of the North Atlantic Oscillation on precipitation across Greenland. *J. Geophys. Res. Atmos.* **113**, 9227 (2008).

[64] Bjørk, A. A. et al. Changes in Greenland’s peripheral glaciers linked to the North Atlantic Oscillation. *Nat. Clim. Change* **8**, 48–52 (2017).

[65] Ramos Buarque, S. & Salas y Melia, D. Link between the North Atlantic Oscillation and the surface mass balance components of the Greenland Ice Sheet under preindustrial and last interglacial climates: a study with a coupled global circulation model. *Climate* **14**, 1707–1725 (2018).

[66] Brils, M., Kuipers Munneke, P. & van den Broeke, M. R. Spatial response of Greenland’s firn layer to NAO variability. *J. Geophys. Res. Earth Surf.* **128**, e2023JF007082 (2023).

[67] Mosley-Thompson E., Readinger C. R., Craigmile P., Thompson L. G. & Calder C. A. Regional sensitivity of Greenland precipitation to NAO variability. *Geophys. Res. Lett.* **32**, 024776 (2005).

[68] Kjær, K. H. et al. Glacier response to the Little Ice Age during the Neoglacial cooling in Greenland. *Earth-Sci. Rev.* **227**, 103984 (2022).

[69] Brooks J. P., Larocca L. J., Axford Y. L. Little Ice Age climate in southernmost Greenland inferred from quantitative geospatial analyses of alpine glacier reconstructions. *Quaternary Sci. Rev.* **293**, 107701 (2022,).

[70] Osman, M. B. et al. Abrupt Common Era hydroclimate shifts drive west Greenland ice cap change. *Nat. Geosci.* **14**, 756–761 (2021).

[71] Zhao, B. Y. et al. Prolonged drying trend coincident with the demise of Norse settlement in southern Greenland. *Sci. Adv.* **8**, eabm4346 (2022). 35319972 10.1126/sciadv.abm4346 PMC8942370

[72] Pöppelmeier, F., Jeltsch-Thommes, A., Lippold, J., Joos, F. & Stocker, T. F. Multi-proxy constraints on Atlantic circulation dynamics since the last ice age. *Nat. Geosci.* **16**, 349–356 (2023). 37064010 10.1038/s41561-023-01140-3 PMC10089918

[73] Smith, D. M. et al. Mitigation needed to avoid unprecedented multi-decadal North Atlantic Oscillation magnitude. *Nat. Clim. Change* **15**, 403–410 (2025).

[74] Mitevski I., Lee S. H., Vecchi G., Orbe C., Polvani L. M. More positive and less variable North Atlantic Oscillation at high CO2 forcing. *npj Clim. Atmos. Sci.* **8**, 171 (2025).

[75] Kucera, M. et al. Deep drilling in the Baffin Bay for marine sediment records of Greenland Ice Sheet collapse during past warm intervals, Cruise No. MSM111, 02.09. - 04.10.2022, Reykjavik (Iceland) - St. John’s (Canada). Bonn: Begutachtungspanel Forschungsschiffe. Report No.2195-8483 (2023).

[76] Mollenhauer, G., Grotheer, H., Gentz, T., Bonk, E. & Hefter, J. Standard operation procedures and performance of the MICADAS radiocarbon laboratory at Alfred Wegener Institute (AWI), Germany. *Nucl. Instrum. Methods Phys. Res. Sect. B Beam Interact. Mater. At.* **496**, 45–51 (2021).

[77] Blaauw, M. Methods and code for ‘classical’ age-modelling of radiocarbon sequences. *Quat. Geochronol.* **5**, 512–518 (2010).

[78] Pearce, C., Özdemir, K. S., Forchhammer Mathiasen, R., Detlef, H. & Olsen, J. The marine reservoir age of Greenland coastal waters. *Geochronology* **5**, 451–465 (2023).

[79] Tjallingii R., Röhl U., Kölling M. & Bickert T. Influence of the water content on X-ray fluorescence core-scanning measurements in soft marine sediments. *Geochem. Geophys. Geosyst.* **8**, 1393 (2007).

[80] Weltje, G. J. & Tjallingii, R. Calibration of XRF core scanners for quantitative geochemical logging of sediment cores: theory and application. *Earth Planet. Sci. Lett.* **274**, 423–438 (2008).

[81] Stalling D., Westerhoff M., Hege H.-C., Hansen C. & Johnson C. The visualization handbook. (Elsevier, 2005).

[82] Bartels, M. et al. Atlantic Water advection vs. glacier dynamics in northern Spitsbergen since early deglaciation. *Climate* **13**, 1717–1749 (2017).

[83] Weiser, J., Titschack, J. & Hebbeln, D. The deglaciation of Upernavik trough, West Greenland, and its Holocene sediment infill: processes and provenance. *Boreas* **52**, 314–340 (2023).

[84] Harris et al. Array programming with NumPy. *Nature* **585**, 357–362 (2020). 32939066 10.1038/s41586-020-2649-2 PMC7759461

[85] Born, A., Imhof, M. A. & Stocker, T. F. An efficient surface energy–mass balance model for snow and ice. * Cryosphere* **13**, 1529–1546 (2019).

[86] Zolles, T. & Born, A. Sensitivity of the Greenland surface mass and energy balance to uncertainties in key model parameters. * Cryosphere* **15**, 2917–2938 (2021).

[87] Tedesco, M. & Fettweis, X. Unprecedented atmospheric conditions (1948–2019) drive the 2019 exceptional melting season over the Greenland ice sheet. * Cryosphere* **14**, 1209–1223 (2020).

[88] Faust, J. C. & Kucera M. X-ray fluorescence (XRF) core scanner raw data of sediment core GeoB25206-2, MARIA S. MERIAN cruise MSM111. (PANGEA, 2026).

[89] Faust, J. C. et al. CT raw data (DICOM format) of sediment core GeoB25206-2, MARIA S. MERIAN cruise MSM111. 2026.

[90] von Dobeneck T., Kucera M., Faust J. C. Electrical conductivity, porosity and density of sediment core GeoB 25206-2. 2025.

[91] von Dobeneck T., Kucera M., Faust J. C. Magnetic susceptibility of sediment core GeoB25206-2. 2025.

[92] Faust J. C. et al. Visual core description and line scanner images of sediment core GeoB25206-2, MARIA S. MERIAN cruise MSM111. 2026.

[93] Steenfelt A. Geochemical atlas of Greenland - West and South Greenland. (Danmarks og Grønlands Geologiske Undersøgelse Rapport, 2001).

[94] Hurrell J. W., Kushnir Y., Ottersen G. & Visbeck M. An overview of the North Atlantic Oscillation. *The North Atlantic Oscillation: Climatic Significance and Environmental Impact*. pp. 1–35 (American Geophysical Union, 2003).

