# Supplementary material for: Persistent influence of the North Atlantic Oscillation on Late Holocene hydroclimate in southwestern Greenland
Source: Nat Commun. 2026 Aug 21;17:8817. doi: 10.1038/s41467-026-77084-0 (PMC13498579; doi:10.1038/s41467-026-77084-0)
Supplement: Supplementary file 1 [file 41467_2026_77084_MOESM1_ESM.pdf]

Supplementary Information

Persistent influence of the North Atlantic Oscillation on Late Holocene hydroclimate in southwestern Greenland

Faust et al.

Sediment Properties

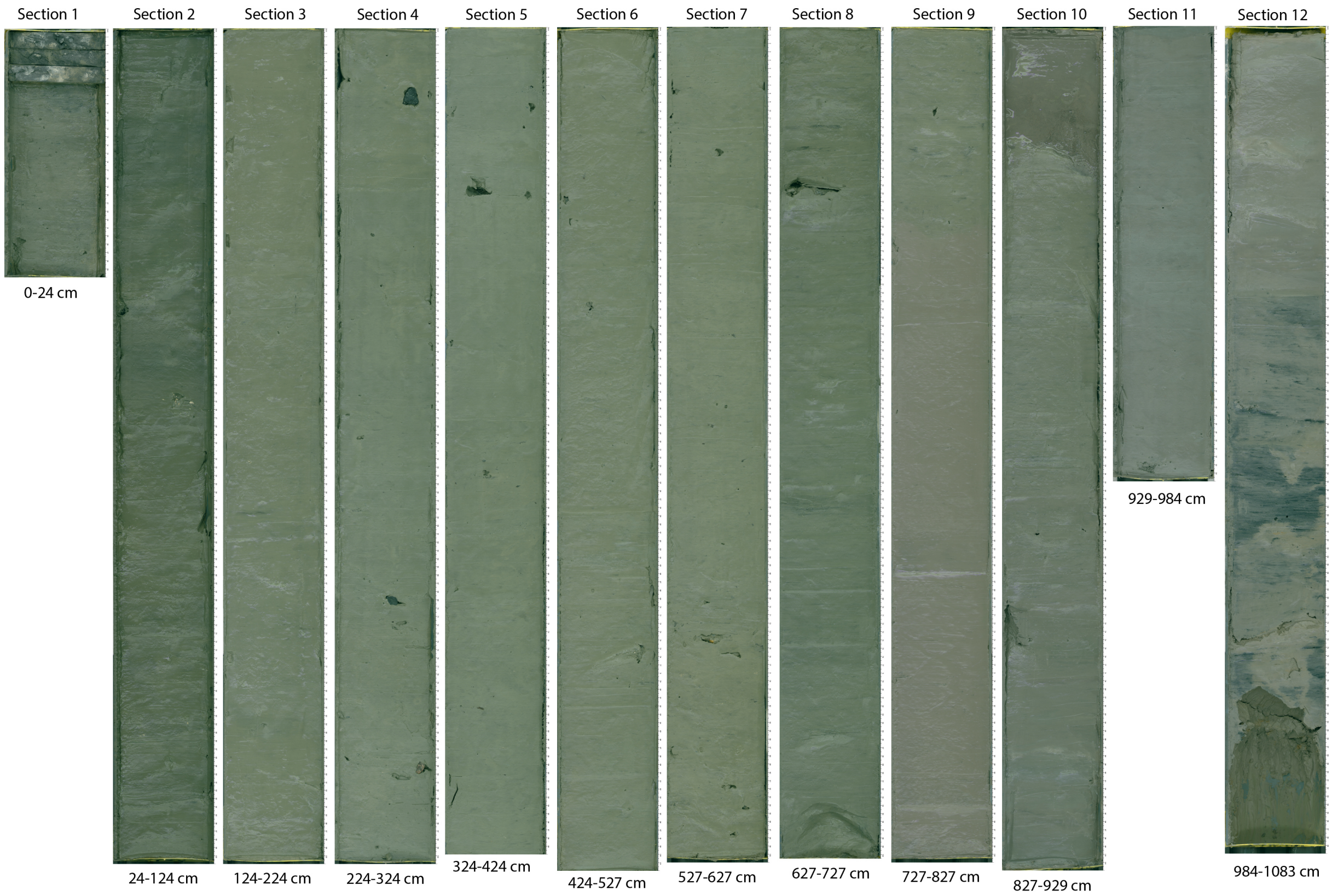

Supplementary Figure 1: Sediment core GeoB25206-2 colour images. The visual inspection of the gravity core GeoB25206-2 shows that the majority of the core, from top to about 750 cm, consists of compact olive-grey fine-grained mud. From 750 cm, GeoB25206-2 shows a transitional colour change from olive-grey to pinkish grey. This interval is intercepted at 827 cm by an about 13 cm thick water rich olive-grey sand layer which is cut off at about 840 cm by a layer of olive-grey fine-grained mud with a downcore colour change to pale red. The grey sediment with a reddish hue is present until the end of the core and large dark-grey patches appear from 1017 cm downcore. Visual core description and high-resolution sediment core line scanner images of the sediment core GeoB25206-2 are available on PANGEA (https://doi.pangaea.de/10.1594/PANGAEA.983000).

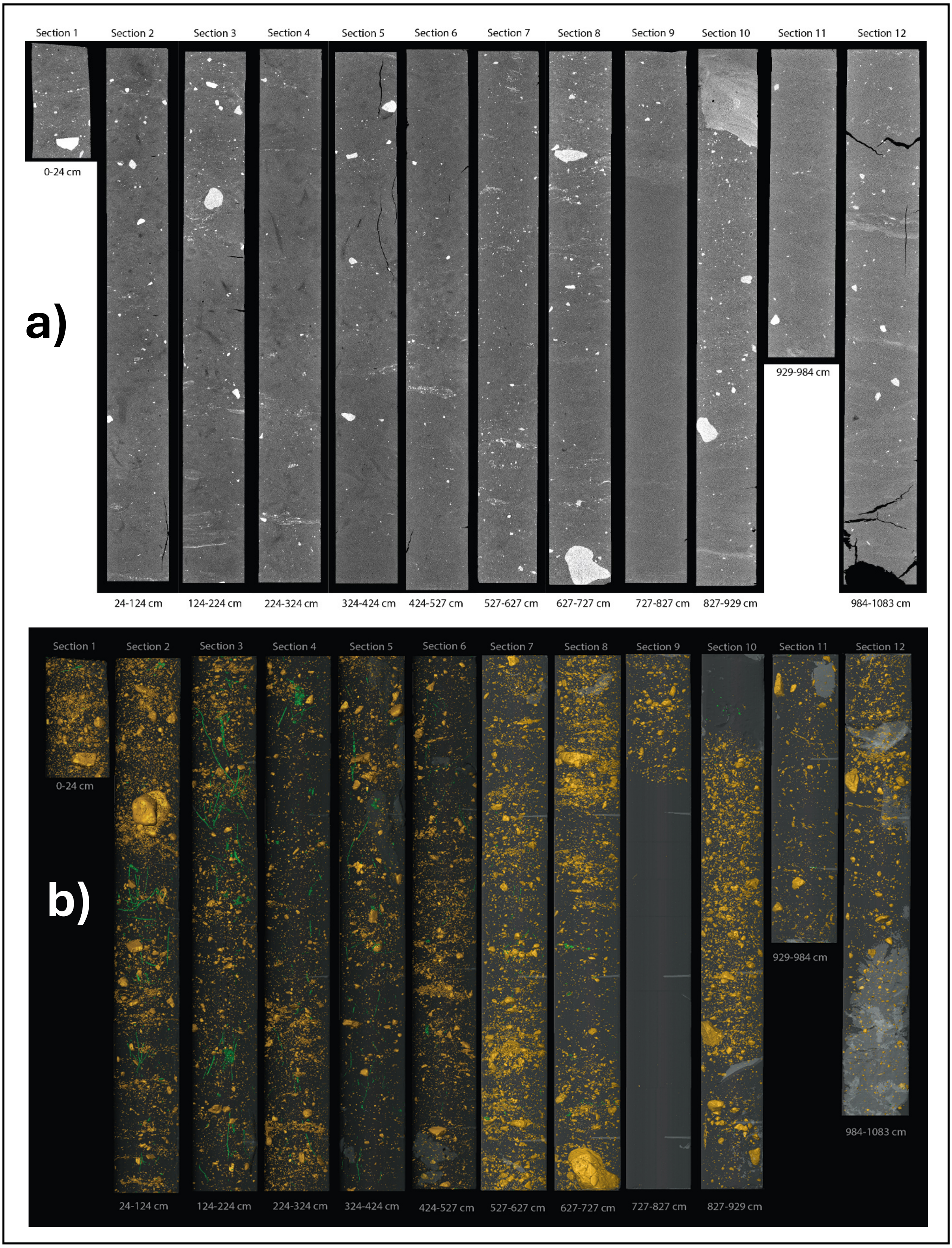

Supplementary Figure 2: Sediment core GeoB25206-2 CT scan grey scale images (a) and 3D reconstructions of IRD grains (yellow) and bioturbation (green) (b).

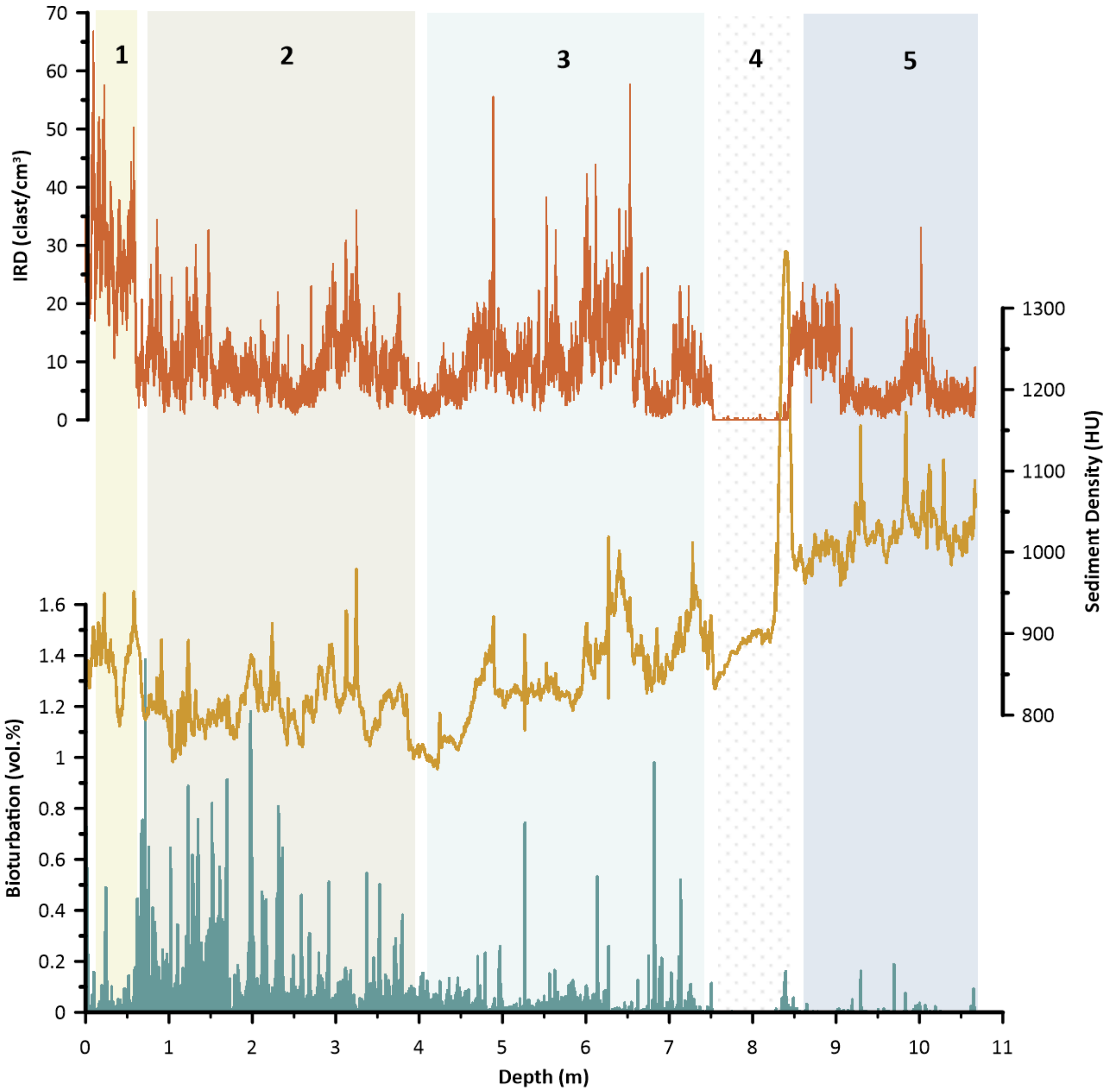

Supplementary Figure 3: In comparison to the superficial visual core description, CT scanning reveals detailed information about the sediment core's internal structure, helping to determine the genesis of the sediment record. Changes in bioturbation, sediment density and IRD concentration subdivide the sediment record into five lithological units.

Throughout the core, the amount of IRD range between 0-67 clasts/cm^3 with an average of 10 clasts/cm^3. Highest IRD numbers (11-67 clasts/cm^3) and lower bioturbation occurs at the top of the core, between 0-60 cm. At about 60 cm, the number of IRD grains drops sharply and the bioturbation intensity increases. Bioturbation stays high until about 250 cm and decreases down to <0.2 vol% at about 400 cm. Besides slightly higher values around 320 cm, IRD numbers are relatively low between 60-400 cm. From 400 cm IRD show an increasing trend until about 650 cm from where the number of IRD grains decrease again until about 750 cm. The sediment layer between 750 cm and 840 cm, unit four (grey dotted area), shows no bioturbation and no IRD and is interpreted as a turbiditic event (see also supplementary Figures 1 and 2). Bioturbation stays very low until the end of the core while the IRD record shows two layers (between 840-910 cm and 980-1010 cm) with >10 IRD clasts/cm^3.

Sediment density varies in accordance with the IRD record until about 650 cm. From 650-827 cm sediment density increases and stays relatively high until the end of the core. A pronounced density peak is found at about 835 cm at the position of a water rich sand layer (see supplementary Figure 2).

Chronology

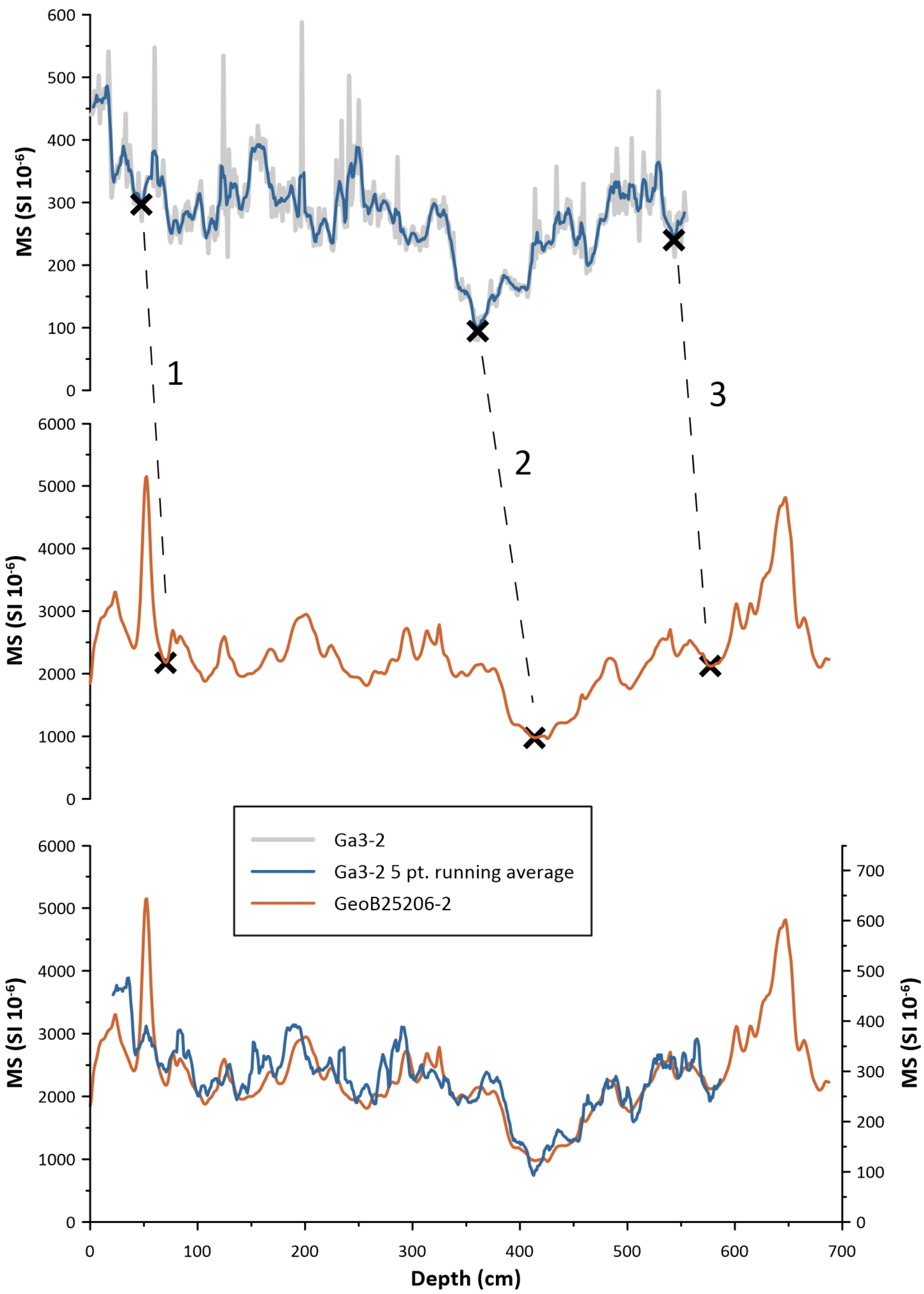

Supplementary Figure 4: Magnetic susceptibility (MS) record of the of Ga3-2 (Nørgaard-Pedersen and Mikkelsen, 2009) with a 5pt. running average overlay compared to MS record (first 700 cm) of the GeoB25206-2. Based on the Ga3-2 age model, sedimentation rate is almost linear below ~600 cm. Therefore, only three anchor points (marked by black crosses) were used to achieve alignment. Both depicted MS records refer to the sediment sequence and therefore largely coincide in relative terms. The deviant scaling of MS logs is therefore believed to have technical and not sedimentological reasons. Magnetic susceptibility data of sediment core GeoB25206-2 are available on PANGEA (https://doi.org/10.1594/PANGAEA.983992).

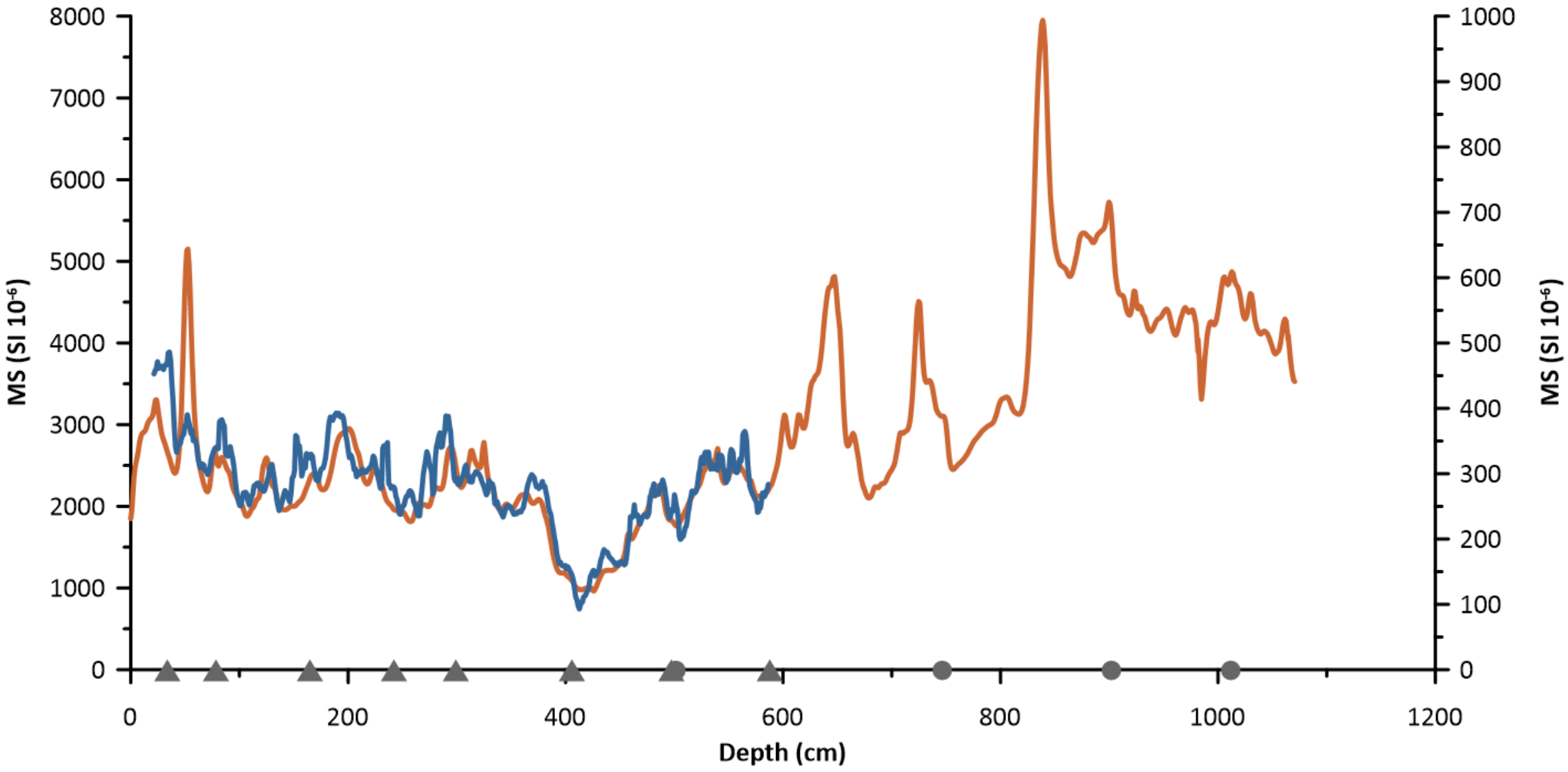

Supplementary Figure 5: Depth alignment of the Ga3-2 magnetic susceptibility record (5 pt. running average) (Nørgaard-Pedersen and Mikkelsen, 2009) and the entire GeoB25206-2 record. Depth of the AMS radiocarbon dates used for the GeoB25206-2 age-depth model (supplementary Table 1, Figure 6) are denoted by grey triangles (Ga3-2) and grey dots (GeoB25206-2).

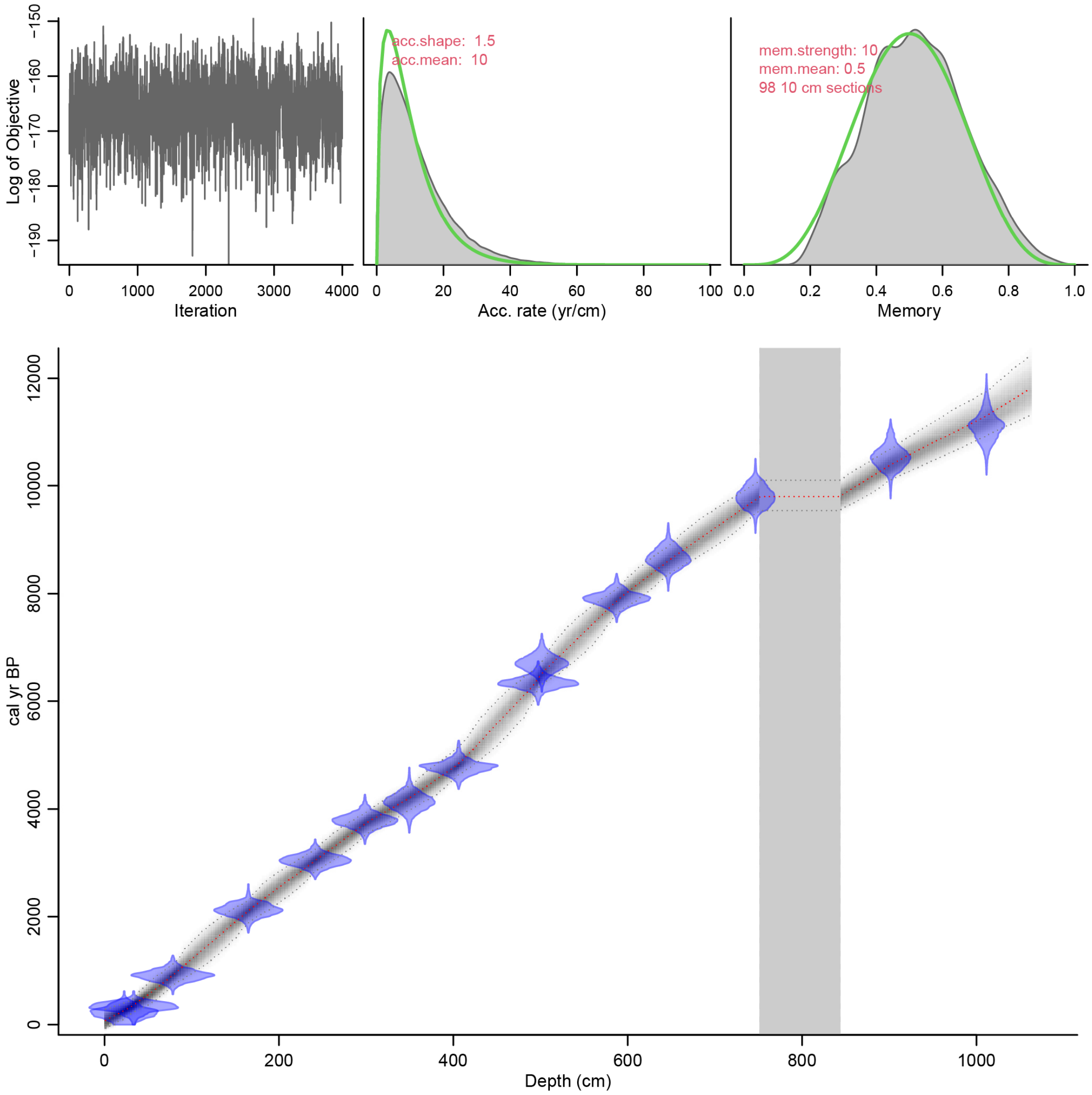

Supplementary Figure 6: The age-depth model of GeoB25206-2, Bacon output (Blaauw, 2010). AMS Radiocarbon ages were calibrated using the MARINE20 curve with reservoir correction of -74 ±24 years (Pearce et al., 2023). The upper panels depict the Markov Chain Monte Carlo (MCMC) iterations (left; good runs show a stationary distribution with little structure among neighbouring iterations), the prior (green curves) and posterior (grey histograms) distributions for the accumulation rate (middle panel) and memory (right panel). The bottom panel shows the calibrated ^{14}C dates (transparent blue) and the age-depth model (darker greys indicate more likely calendar ages; grey stippled lines show 95% confidence intervals; the red curve shows a single 'best' model based on the mean age for each depth).

The age-depth model indicates that the base of the sediment core GeoB25206-2 extends back to almost 12 ka cal. BP. Moreover, sedimentation rate is relatively constant, about 90 cm/ka, until ~4.9 ka (416 cm). From this point, sedimentation rate decreases slightly to an average of about 69 cm/ka until 9.7 ka (752 cm). The sediment unit from 752-843 cm (vertical grey shadow), which shows no bioturbation, no IRDs and which is partly sandy, water rich and shows high density values (supplementary Figures 1, 2, 3 and 4) is interpreted as a turbiditic event. Therefore, this depth interval is assumed to be deposited in less than a year. With an average of 107 cm/ka, highest sedimentation occurs below the mass-wasting deposit (>9715 cal yr BP) until the end of the core.

Numerical Modelling

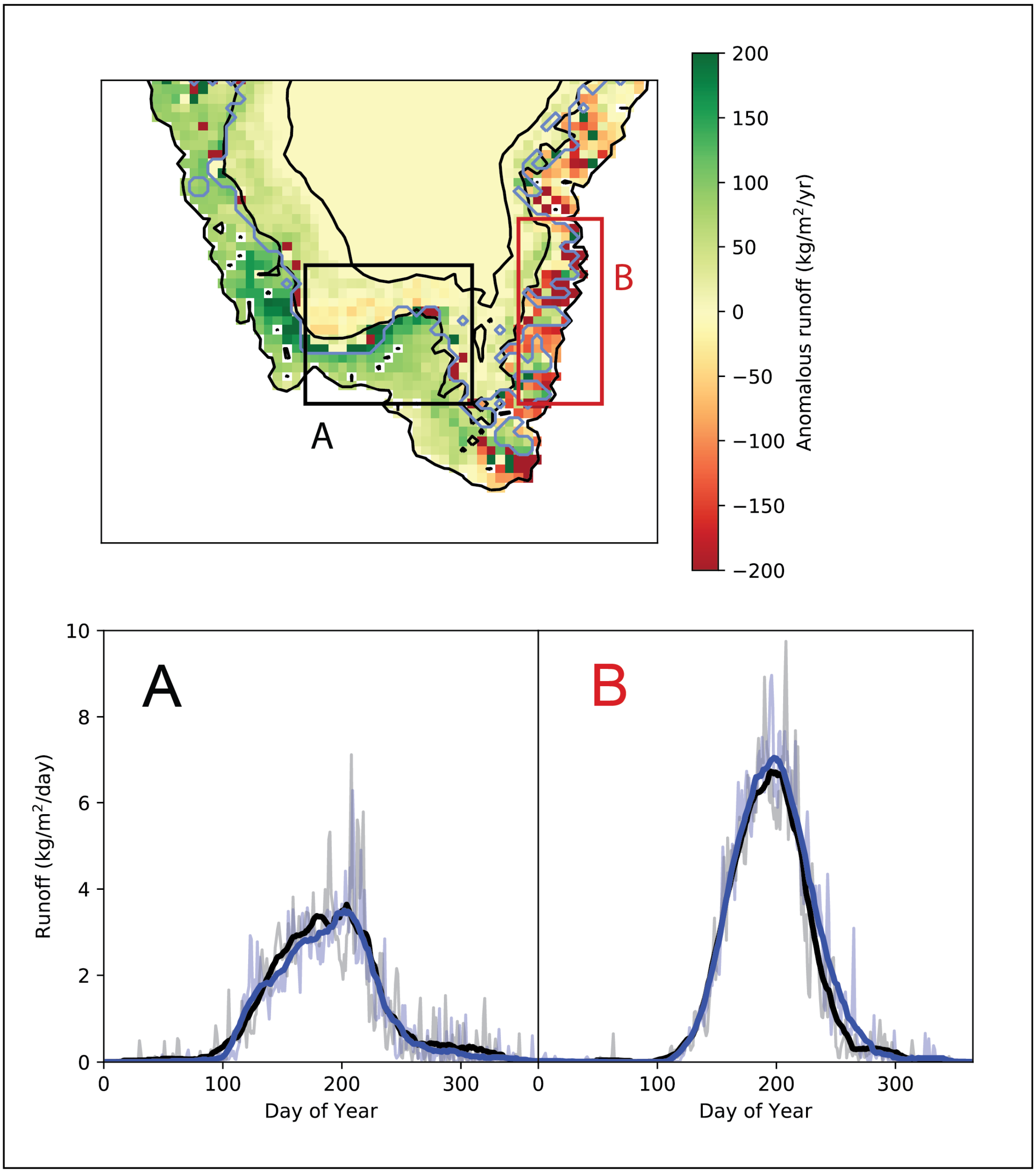

Supplementary Figure 7: Upper figure: South Greenland regional anomalous runoff 'NAO+ minus NAO-' as calculated by BESSI (Born et al., 2019; Zolles and Born, 2021) and observed ice sheet margin (blue line, Luetzenburg et al., 2025). Lower figure: Average runoff from region A and B (upper figure), for NAO+ (black) and NAO- (blue).

Sensitivity analysis of smoothing and correlation parameters

To assess the robustness of correlations between the Niobium (Nb) record and the comparison datasets, time series were interpolated pairwise to a common, evenly spaced time grid (*dt* = 20 years) over their respective overlapping intervals and smoothed using moving-average filters with window lengths between 50 and 250 years.

First, correlations were evaluated over the full overlapping interval (i.e., without applying a moving-window approach) using normalized cross-correlation with zero lag (supplementary Figure 8). For each smoothing window, the zero-lag, normalized cross-correlation coefficient between the Nb record and each comparison dataset was calculated (using xcorr in MATLAB), and the resulting correlation coefficients were plotted as a function of smoothing window length.

Second, moving-window correlations were computed using window lengths of 1000 and 1500 years and a step size of 50 years (supplementary Figure 9). Pearson correlation coefficients were used as the primary metric, and Spearman rank correlations were additionally calculated to assess robustness with respect to non-linearity and outliers.

The sensitivity analysis shows that correlation strength increases systematically with increasing smoothing window, indicating that the relationships are primarily expressed at centennial to millennial timescales. Variations in ***dt*** have only minor effects, primarily influencing shorter records where high-frequency variability is more pronounced.

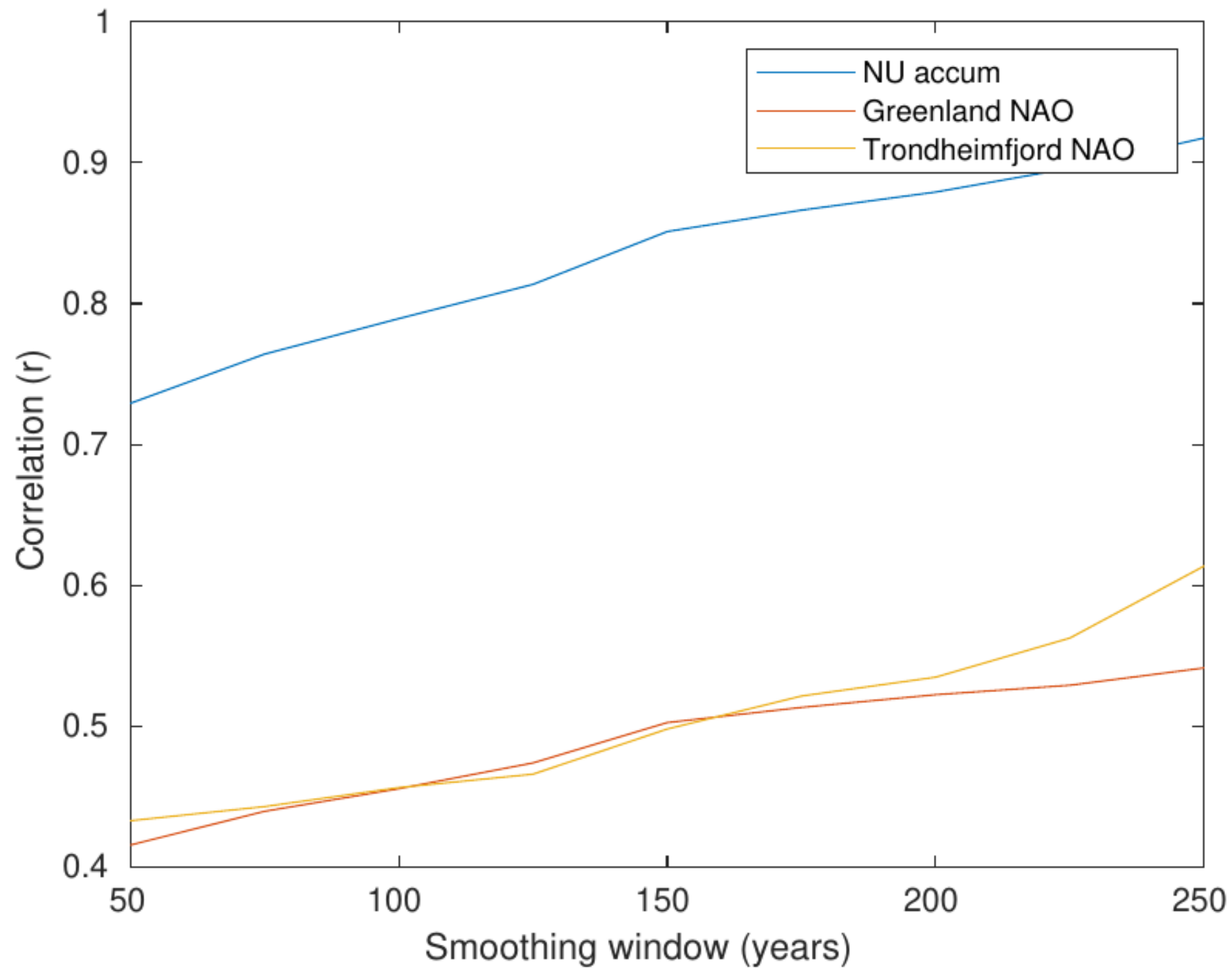

Figure 8: Correlation coefficients (r) as a function of smoothing window for each dataset shown in Fig. 5. Correlations were computed pairwise over the time interval overlapping with the Niobium record, after interpolation to a common time grid with a step size of *dt* = 20 years. For all datasets, correlation increases systematically with increasing smoothing window, indicating that the coupling between the proxies is primarily expressed at centennial to millennial timescales, while high-frequency variability is dominated by noise or local processes.

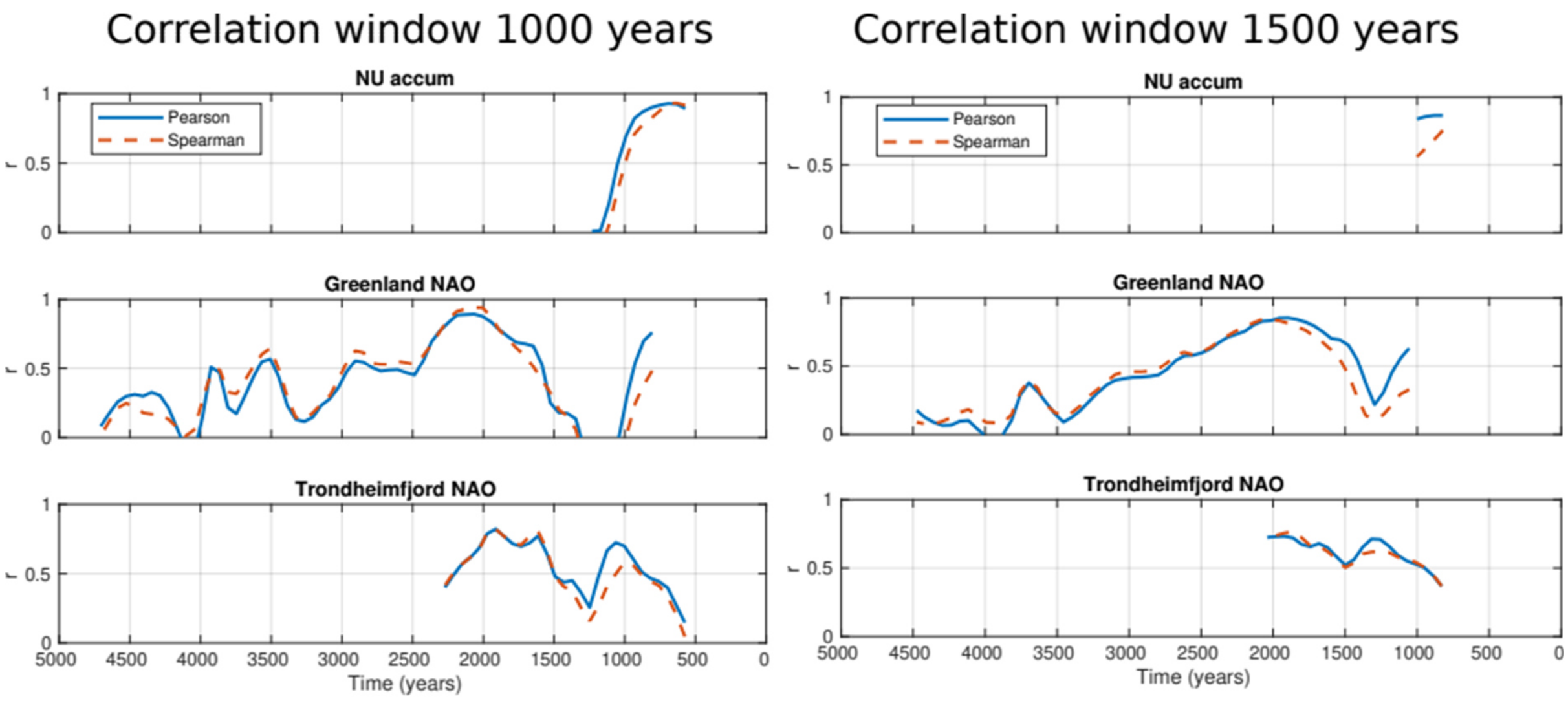

Supplementary Figure 9: Moving-window correlations between the Niobium record and the datasets shown in Fig. 5, computed over their respective overlapping intervals. Correlation windows of 1000 years (left column) and 1500 years (right column) were applied to assess the persistence of the relationship between NAO variability and Nb (precipitation) on multi-centennial to millennial timescales. All correlations were computed using an interpolation step of dt = 20 years, a smoothing window of 150 years, and a step size of 50 years. Pearson (blue, solid) and Spearman (red, dashed) correlation coefficients show very similar temporal patterns across all datasets, indicating that the observed relationships are robust and not driven by outliers or strictly linear dependencies. Increasing the window length results in smoother correlation curves and reduced variability, while preserving the overall temporal structure.

References

Blaauw, M., 2010, Methods and code for 'classical' age-modelling of radiocarbon sequences: Quaternary Geochronology, v. 5, no. 5, p. 512-518.

Born, A., Imhof, M. A., and Stocker, T. F., 2019, An efficient surface energy–mass balance model for snow and ice: The Cryosphere, v. 13, no. 5, p. 1529-1546.

Luetzenburg, G., Korsgaard, N. J., Deichmann, A. K., Socher, T., Gleie, K., Scharffenberger, T., Fahrner, D., Nielsen, E. B., How, P., Bjørk, A. A., Kjeldsen, K. K., Ahlstrøm, A. P., Fausto, R. S., 2025, "PROMICE-2022 Ice Mask", GEUS Dataverse, V3

Nørgaard-Pedersen, N., and Mikkelsen, N., 2009, 8000 year marine record of climate variability and fjord dynamics from Southern Greenland: Marine Geology, v. 264, no. 3-4, p. 177-189.

Pearce, C., Özdemir, K. S., Forchhammer Mathiasen, R., Detlef, H., and Olsen, J., 2023, The marine reservoir age of Greenland coastal waters: Geochronology, v. 5, no. 2, p. 451-465.

Zolles, T., and Born, A., 2021, Sensitivity of the Greenland surface mass and energy balance to uncertainties in key model parameters: The Cryosphere, v. 15, no. 6, p. 2917-2938.
