# Supplementary material for: Persistent influence of the North Atlantic Oscillation on Late Holocene hydroclimate in southwestern Greenland
Source: Nat Commun. 2026 Aug 21;17:8817. doi: 10.1038/s41467-026-77084-0 (PMC13498579; doi:10.1038/s41467-026-77084-0)
Supplement: Supplementary file 2 [file 41467_2026_77084_MOESM2_ESM.pdf]

Description of the Additional Supplementary Files

Title: Supplementary Data 1
Description: "Radiocarbon data"

Title: Supplementary Data 2
Description: "CT core scanning data"

Title: Supplementary Data 3
Description: " X-ray fluorescence (XRF) core scanning data"

Title: Supplementary Data 4
Description: " Data used to derive the moving-window correlations"
