# Supplementary material for: Persistent influence of the North Atlantic Oscillation on Late Holocene hydroclimate in southwestern Greenland
Source: Nat Commun. 2026 Aug 21;17:8817. doi: 10.1038/s41467-026-77084-0 (PMC13498579; doi:10.1038/s41467-026-77084-0)
Supplement: Supplementary file 3 [file 41467_2026_77084_MOESM7_ESM.pdf]

nature portfolio

Peer Review File

Persistent influence of the North Atlantic Oscillation on Late Holocene hydroclimate in southwestern Greenland

Corresponding Author: Dr Johan Faust

This file contains all reviewer reports in order by version, followed by all author rebuttals in order by version.

Parts of this Peer Review File have been redacted as indicated to remove third-party material.

Version 0:

Reviewer comments:

Reviewer #1

(Remarks to the Author)
This manuscript presents a well-written and concise study showing strong potential for NAO reconstruction. The quality of the age model is particularly impressive and lends robustness to the interpretations, even though the methods applied to the sedimentary archives are relatively simple. Overall, the study is of high quality and provides valuable insights.

I have some concerns regarding the interpretation of the observed trends and their amplitudes throughout the record. It is not entirely clear whether these trends can be attributed solely to NAO variability, or whether they may also reflect modulation by (for example) longer-term sea surface temperature (SST) anomalies. Given that one of the reconstructions is derived from a fjord setting, oceanic influences could plausibly play an important role. The authors should discuss the potential contribution of long-term SST variations in shaping these trends. It is also noteworthy that the magnitude of the reconstructed NAO variability appears larger than that reported in previous reconstructions (e.g., Ortega et al., 2015), which generally do not show such pronounced long-term trends. A discussion comparing the amplitude and trends in this reconstruction with existing NAO reconstructions would strengthen the interpretation. Any long-term SST implications possible? I must admit that the comparison with reconstructed NAO appear quite convincing, but again I am wondering how the oceanic SSTs play a role in their fluctuations.

Furthermore, the interpretation of a drier LIA seems somewhat inconsistent with evidence for glacier expansion during the LIA in Greenland and the Canadian Arctic (e.g., Miller et al., 2012; Jomelli et al., 2016, Young et al. 2015), which typically implies cooler and possibly wetter conditions. The authors may wish to reconcile this apparent discrepancy within the broader climatic context.

Minor points :

No labelling (A,B, C) in figure 5

Plaese add R coefficient and p values in figure 5 (taking autocorrelation into account).

Please provide the spectral content of your record in the supplemental information.Any frequencies linked to long-term NAO and/or SSTs as well as external forcing?

Lines 245-249 : please rephrase

Reviewer #2

(Remarks to the Author)
Review of
"Persistent influence of the North Atlantic Oscillation on Late Holocene hydroclimate in southwestern Greenland"
by J.C. Faust et al.

With interest have I read this manuscript, which is generally well writen and clear. I was triggered to review this paper by the last sentence of the abstract, which state that the Greenland Ice Sheet (GIS) may grow locally due to future changed precipitation patterns.

In essence, the manuscript consists of two components. The first component is the description and analysis of the presented proxy record from southern Greenland. This component is well and clearly written. However, I have to admit that paleorecords are out of my expertise, so opinions of other reviewers on this component should prevail over my opinion. The second component is the interpretation and implications of the results from the proxy records. Again, this part is well written, however, to my opinion the core interpretations of the manuscript are currently not sufficiently proven. These interpretations need either more evidence, or otherwise, must be rewritten in a next version of this manuscript.

The first interpretation is that the proxy record, through the Nobium concentration, demonstrates that local precipitation is, since 5 ky BP, is governed by NAO. This is demonstrated in Figure 5, which is visually rather convincing. However, next to this qualititve match, the authors should provide quantitative evidence of this NAO steering, hence, what is the correlation between the Nobium record and the various NAO index proxies. It might be that some temporal smoothing is needed to get reasonable correlations - I leave that to the authors to figure out and motivate choices on this matter.

The second interpretation in line 279-280, namely: "Collectively, our results demonstrate that southern Greenland hydroclimate and glacier dynamics were strongly modulated by NAO variability"
Figure 5 (and additional statistics will) show(s) the link between southern Greenland precipitation and NAO, and Figures 2 and 3 show the link between ice dynamics and climate, hence I do not see any (articulated) demonstration that glacier dynamics is modulated by NAO. As I see the data, as presented now, there is a clear and well known relation between temperature, precipitation and ice sheet dynamics. If the authors think NAO also steers ice dynamics, they should provide more evidence, for example that any/all observed variables presented in Figure 2 correlate with NAO proxies. If this evidence is not there or cannot be demonstrated, this interpretation cannot be hold.

The third and last interpretation is that the proxy data rise the possibility that the projected increase of NAO will allow for regional expansion of the Greenland Ice Sheet. Next that I am not convinced that it is demonstrated that the NAO stir local glacier dynamics, I see a fundamental problem. The projection that the atmosphere will exhibt in the future a preference for a NAO+ state is derived from Earth System Model (ESM) simulations. At the same time the surface mass balance (SMB) and ice sheet projections that project that the GIS will shrink, are based on ESMs too. Hence, if the possibility of the authors is real, then either publications on the future SMB must have overlooked that this region in southern Greenland will have an increasing SMB, or the SMB projections (based on regional climate model (RCM) simulations) all have used ESM realisations in which the NAO does not increase as is anticipated by the authors. By chance, I have such a future SMB projection at hand, and this realization has both a clearly increasing NAO. I've checked the modelled SMB for depicted region with increased SMB in Figure 4-4. In this realization I have at hand, the precipitation over the ice sheet does not significantly increase while melt and runoff increase considerably - hence, also here the Greenland Ice Sheet will retreat. It could be, of course, that the realization that I have at hand is one-off. Still, it is to the authors to demonstrate that either the available SMB projections over Greenland do not have this NAO+ effect which is prominent in the whole ESM ensemble, or that this local SMB increase is there in the RCM projections, but nobody has noticed before.
In the end, the conclusion that the GIS might locally grow in the future is bold (and might give rise to the erroneous interpretation that GIS will florish in a future warmer climate), and hence cannot be based on the interpretation of proxies only, especially when there are ample projections available to evaluate the validity/likelyhood of this claim.
Subsequently, if the authors do not, or cannot provide this additional support for the conclusion that this part of the GIS might expand (slightly) in the future, the conclusion on how an increasing NAO influences the sourthern GIS must be reformulated.

Minor comments:
L33: Please use Greenland Ice Sheet, or Greenland Ice Sheet and Ice Caps, or similar, but not this - it is also an one-off.
Figure 4: Please specify which time-period is covered by the displayed data, and which NAO definition is used.
Figure S7: Please display the ice sheet edge in model data. Furthermore, it seems that there is mostly a tundra-runoff response. Why is there very little response in the runoff from the ice sheet?

Version 1:

Reviewer comments:

Reviewer #1

(Remarks to the Author)
I am overall satisfied with the authors` responses. One last comment: Did they authors try to compare their Nb record to reconstructed AMOs (Mann et al. 2009, Wang et al. 2017 & Lapointe et al. 2020)?

Best regards

Reviewer #2

(Remarks to the Author)
2nd Review of " Persistent influence of the North Atlantic Oscillation on Late Holocene hydroclimate in southwestern

Greenland"

In my first review I raised 3 main concerns, and the authors have addressed these. However, I must admit I'm not fully convinced that my concerns are sufficiently covered.

The first concern was the relations shown in Figure 5 were only demonstrated in words, not in numbers. This is sufficiently addressed by the authors. Nonetheless, it would be good if the authors include in the supplementary materials the smoothed version of the records which were used to derive the running correlations shown in Figure S9.

The second concern was that Figure 2&3 does not show - as far as I can see - any relation between NAO and ice sheet/glacier dynamics. I asked the authors either to 'articulate' this relation, that is, describe (and prove) clearly in the manuscript, or refrain of making conclusions. The authors choose to 'soften their claim', thus did neither one of the two, and that is a pity. Even though it is demonstrated that NAO variability modulates the precipitation amounts in this region of Greenland, that does not necessarily imply that glacier dynamics is considerably influenced by this variability. The amplitude or period might be too short to have a measurable impact, after all an ice sheet responds slow. Yes, it may have influenced local glaciers and the ice sheets surface mass balance, but no evidence has been provided that ice sheet dynamics has been altered by NAO variability. So again, prove that that there is a NAO signal in local ice sheet dynamics, or refrain of claiming this.

The third concern was the evidence for the conclusion that with future increasing NAO conditions, the ice sheet might locally grow. The authors mention in their rebuttal results of fast and simplified RCM (BESSI), driven by CESM1 data. Although there is indeed some green (increasing SMB) in panel 14b of this figure, there is still, also on the ablation zone in the region of interest, dark red, thus sharply decreasing SMB). So, there is indeed compensation, in these simulations, by increasing precipitation, but as I see it, there is still no offset - and if there is so, the authors could easily demonstrate that from their results by integrating the SMB difference over the drainage basin of the glaciers that are presumed to grow. Besides, that, using a fast and simplified RCM (intended for long (>100 ky) ice sheet simulations) to prove the point is not the optimal model, as there also 'normal' RCM data available for this time scale. Again, I am not aware that any of the RCM projections published (or shared unpublished) give the possibility for an expanding ice sheet in the south.

Concluding, in my opinion the model and proxy data doesn't provide enough evidence that NAO influence the ice sheet dynamics and may give rise to a local expansion of the ice sheet. If I was the author of this paper, I would refrain thus from writing down both conclusions, even in a "may be"-form.

Additionally, I realized that the major local change that could impact the GrIS, if happening, is a significant change in the AMOC. The impact of the cooling ocean south-east of Greenland is a reduced precipitation and a reduced increase of the temperature; and overall might slow down the mass loss of the GrIS. There is, as far as I know, limited published work on it, but existing literature does demonstrate that changes in the ocean temperatures have a distinct impact on the glaciers of Iceland, which is upstream of south-east Greenland. Anyhow, what I wish to convey: the best projections of the upcoming century come from ESMs and RCMs. These models aren't perfect, and past climate reconstructions through proxy data can help to find missing climate processes. In this case, a future changing NAO is (well) represented by the ESMs, and the authors fail to demonstrate that the modelled resulting impact on the GrIS, namely marginal, is incorrect.

Lastly, the authors mention never variability in the AMOC, even though this has a major impact on the ice sheet. Yes, there is no indication that during the considered period the AMOC changed significantly, but it would be better if the authors mention that changes in the AMOC is unlikely a cause of the changes in the time period of interest, as there is no evidence of any significant changes in the AMOC during the past 5 ky (e.g. Pöppelmeier et al, 2023, NatGeo, https://doi.org/10.1038/s41561-023-01140-3).

Minor comments:
Figure 2:
It might be visually helpful to add dashed colored lines to see quantitative changes better. Like a brown dashed line for 700 (or 750) HU for the sediment density line (just below/trough the minimum values @ 5 ky BP) and a red-brown dashed line at 0 clast/cm3 for the IRD.

L447: "All data of sediment core GeoB250206-2 supporting the findings of this study are..." Please rewrite to remove the suggestion that the core parts that weren't supporting the findings of this study are not available on PANGAEA

Version 2:

Reviewer comments:

Reviewer #2

(Remarks to the Author)
3rd review of
Persistent influence of the North Atlantic Oscillation on Late Holocene hydroclimate in southwestern Greenland

The remaining two concern in the 2nd review were:

• Figure 2&3 does not show - as far as I can see - any relation between NAO and ice sheet/glacier dynamics,
• The evidence for the conclusion that with future increasing NAO conditions, the ice sheet might locally grow.
The authors extensively discuss these two remaining questions and have adjusted the manuscript.

Regarding the first concern: There is ample proof, also from the results presented in this manuscript, that North Atlantic Oscillation (NAO) influences the precipitation and subsequently the surface mass balance (SMB). Of course, the SMB and glacier/ice sheet dynamics are closely linked. However, the bottleneck in the argument is the question of whether the variability in SMB is large and persistent enough to cause a measurable change in ice sheet dynamics. As the authors correctly point out, Figures 2 and 3 do not directly show a relation between the NAO and ice sheet/glacier dynamics. The four references in the rebuttal demonstrate the relation between NAO and SMB, but not between NAO and ice sheet dynamics. I have also checked the five additional references in the manuscript. Osman et al. (ref. 70) find that past ice cap changes are linked to NAO changes, but they also conclude that this will not be leading in the future as warming simply eliminate all accumulation zones. Zhao et al., Smith et al., Mitevski et al. (refs 71, 72 and 73) do not address ice sheet changes. Kucera (ref. 74) is a field report and I cannot see (but yes, the report is long) where it proves the link between NAO variability and observable ice sheet changes. Therefore, I still do not see any evidence here or in the literature that NAO variability is strong and persistent enough to cause traceable/relevant changes in the ice sheet dynamics.

Regarding the second concern, let us not forget that the initial manuscript included the following sentence: "Surface mass balance (SMB) calculations under NAO+ conditions (Fig. 4) reveal, in accordance with our late Holocene sedimentary data, that in such a scenario southwestern GIS margins will experience localized growth due to cooler, wetter conditions, while closely adjacent sectors will exhibit negative SMB implying enhanced ice loss." The second version read: "Surface mass balance (SMB) calculations under NAO+ conditions (Fig. 4) are consistent with our late Holocene sedimentary data, suggesting that southwestern GIS margins may experience localized growth under cooler, wetter conditions, while adjacent sectors could exhibit negative SMB and enhanced ice loss", and a similar statement is made in the lastest draft. In their rebuttal, the authors state: "While we concede that the phrasing of our original submission may have been ambiguous, we reject the notion that we make claims about a future expansion of the Greenland ice sheet." I agree this claim is not made for the Greenland Ice Sheet as a whole, but it is certainly made for the southwestern GIS. As stated above, there is no evidence of NAO-induced changes in glacier dynamics. There may have been some, but then it was too small to be detectable in the proxies you present.
Furthermore, the best method currently available to project future (southern) Greenland Ice Sheet dynamics is to use dynamically downscaled SMB by regional climate models and run ice sheet models. I have inquired whether any of the RCM outputs available for ISMIP7 may suggest ice sheet or local glacier growth, but this has been ruled out. This ISMIP7 dataset does include ESM forcings with a NAO+ future, as well as numerous MAR simulations. Therefore, if an increase of the SMB is possible locally for an NAO+ future, it would likely be represented in an ISMIP7 realisation. [Again the absence of such a realisation does not prove that it is impossible; that depends on the mean imbalance of the basins divided by the variability of the SMB across realisations - and I do not have those numbers to hand.] As stated in an earlier review, I am wary of making bold guesses about future behaviour that could be misinterpreted.

To leave this discussion and our review-resubmit loop, I propose rephrasing lines 292-299 (in 661466_2_related_ms_13065618_th9g1x.docx) as follows:
"Our results indicate that NAO variability influenced regional hydroclimate, particularly precipitation. This, in turn, affected the local ice-sheet mass balance, in line with other studies that demonstrate a relationship between NAO and SMB[e.g. 70, refs rebuttal]. Our proxy data do not provide evidence that NAO variability subsequently caused changes in glacier and ice sheet dynamics, but neither do they exclude the possibility of small changes. So far, late Holocene..."
and it would be great if the term "small" could be quantified, for example as 'such as local advances of less than a kilometre'

And rephrase 358-368 as follows (removing the last two sentences as they are, in my view, repeating previous sentences. I also don't see how 74, an expedition report, proves anything about the future):
... , some model projections suggest that increasing CO_2 concentrations may favour a more positive and less variable NAO state[71-73, 79, 80]. Surface mass balance (SMB) calculations under NAO+ conditions (Fig. 4) are consistent with our late Holocene sedimentary data. This suggest that the southwestern GIS margins may be more stable in the future, with reduced glacier retreat compared to other areas of Greenland. Further detailed analyses of NAO+ projections could reveal whether increased precipitation could cause localised glacier growth, or whether enhanced ablation by warming remains the leading mass balance process. Moreover, the sharp ...

If the authors are willing to include these or similar reformulations, I have no longer concerns that needs to be addressed. If not, I will leave it to the editor to decide how to proceed.

Minor:
About Figure 2: See the uploaded adjusted Figure as explanation what I meant in the previous review. If adopted, move then the supporting horizontal lines behind the data and, maybe, the sediment density starting at 750 HU (and add the dashed ochre line then at 750 HU).

Open Access This Peer Review File is licensed under a Creative Commons Attribution 4.0 International License, which permits use, sharing, adaptation, distribution and reproduction in any medium or format, as long as you give appropriate credit to the original author(s) and the source, provide a link to the Creative Commons license, and indicate if changes were made.
In cases where reviewers are anonymous, credit should be given to 'Anonymous Referee' and the source.
The images or other third party material in this Peer Review File are included in the article's Creative Commons license, unless indicated otherwise in a credit line to the material. If material is not included in the article's Creative Commons license and your intended use is not permitted by statutory regulation or exceeds the permitted use, you will need to obtain permission directly from the copyright holder.
To view a copy of this license, visit https://creativecommons.org/licenses/by/4.0/

Reviewer #1

Main comments

This manuscript presents a well-written and concise study showing strong potential for NAO reconstruction. The quality of the age model is particularly impressive and lends robustness to the interpretations, even though the methods applied to the sedimentary archives are relatively simple. Overall, the study is of high quality and provides valuable insights.

I have some concerns regarding the interpretation of the observed trends and their amplitudes throughout the record. It is not entirely clear whether these trends can be attributed solely to NAO variability, or whether they may also reflect modulation by (for example) longer-term sea surface temperature (SST) anomalies. Given that one of the reconstructions is derived from a fjord setting, oceanic influences could plausibly play an important role. The authors should discuss the potential contribution of long-term SST variations in shaping these trends.

> *Reply:* *We agree that NAO variability is unlikely to be the sole driver of the observed changes and that additional factors, such as regional SST variability, may also contribute. To support our interpretation, we have added a statistical analysis (Table 1; supplementary information Fig. 8 and 9) demonstrating a robust relationship between our Nb record and existing, independent NAO reconstructions. This analysis does not test the influence of SST directly, but it quantitatively shows that large-scale atmospheric circulation is an important control on the observed variability.*
>
> *Continuous, high-resolution SST reconstructions from coastal southern Greenland covering the full Holocene are currently lacking, which makes it difficult to assess its influence. Moreover, the relationship between SST anomalies and the NAO is complex and not fully understood (e.g. Wang et al., 2004 https://doi.org/10.1175/JCLI-3186.1). In the presented study, we show that changes in the elemental composition of marine sediment core GeoB25206-2 are influenced by local temperature and precipitation changes which are strongly modulated by the large-scale atmospheric circulation pattern. While SST variability may also affect these relationships, a detailed investigation of SST-NAO interactions lies beyond the scope of this study and would require additional proxy data.*

It is also noteworthy that the magnitude of the reconstructed NAO variability appears larger than that reported in previous reconstructions (e.g., Ortega et al., 2015), which generally do not show such pronounced long-term trends. A discussion comparing the amplitude and trends in this reconstruction with existing NAO reconstructions would strengthen the interpretation. Any long-term SST implications possible? I must admit that the comparison with reconstructed NAO appear quite convincing, but again I am wondering how the oceanic SSTs play a role in their fluctuations.

> *Reply:* *We note that our study does not present a new NAO reconstruction, but instead compares our new, independent hydroclimate proxy (niobium) with previously published NAO indices. Therefore, the variability observed in our Nb record should not be interpreted as a direct measure of NAO amplitude. Differences compared to other NAO*

reconstructions (e.g., Ortega et al., 2015) likely reflect proxy-specific sensitivities and regional environmental responses rather than an enhanced magnitude of NAO variability itself.

We agree that oceanic SST variability may influence regional hydroclimate and could contribute to the observed trends. However, a more detailed investigation of SST–NAO interactions would require additional proxy constraints and lies beyond the scope of the present study (see also our response to the 1st question). In our study we show that sediment transport from land is driven by river runoff and relates to precipitation and NAO variability.

Furthermore, the interpretation of a drier LIA seems somewhat inconsistent with evidence for glacier expansion during the LIA in Greenland and the Canadian Arctic (e.g., Miller et al., 2012; Jomelli et al., 2016, Young et al. 2015), which typically implies cooler and possibly wetter conditions. The authors may wish to reconcile this apparent discrepancy within the broader climatic context.

<u>*Reply:*</u> *In the revised manuscript we now added the following sentences to further support our assumption that the LIA was characterized by drier conditions in south Greenland (line 292-297): "Moreover, investigations of lacustrine biomarker data from a lake located 35 km northeast of our coring site revealed a prolong drying trend which peaked in the 16th century during the LIA[71]. Consistent with our interpretation, both studies attribute the regional hydroclimate changes to a long-term shift from predominantly positive NAO conditions during the MCA to predominantly negative NAO conditions during the LIA"*

Past glacial activity is difficult to reconstruct in a continuous manner with a high temporal resolution required to observe glacial activity changes during the LIA. Moreover, glacial response may react with a delay due to the inertia of ice caps, the identification of the response of south Greenland glaciers to short term climate changes, such as the LIA, might therefore be challenging. Similar to the local glacier activity reconstructions in south Greenland, also evidence for glacier expansion during the LIA in Greenland and the Canadian Arctic (e.g., Miller et al., 2012; Jomelli et al., 2016, Young et al. 2015) do not provide an overall consistent picture. Even though some records show large expansion during the LIA, they do not occur at the same time and not during the entire LIA period. Moreover, in the Canadian Arctic a negative NAO (during the LIA) relates to colder conditions (Young et al., 2015; Faust et al., 2016) while, as discussed in the presented study, a negative NAO in south Greenland relates to overall warmer conditions.

<u>Minor points:</u>

No labelling (A,B, C) in figure 5

<u>*Reply:*</u> *We modified figure 5 accordingly*

Please add R coefficient and p values in figure 5 (taking autocorrelation into account).

Reply: In the revised version of the manuscript, we have added quantitative correlation results (Table 1) based on smoothing window correlations (r) computed separately for each dataset shown in Fig. 5. In addition, we include a comprehensive sensitivity analysis in the Supplementary Information (supplementary Figs. 8 and 9), which evaluates the robustness of the correlations with respect to smoothing window length, moving-window size, and interpolation parameters.
We have not included p-values in Fig. 5, as standard significance estimates are problematic for our time series. The records are strongly autocorrelated, and the applied smoothing and moving-window approaches further reduce the number of independent observations and can artificially inflate correlation coefficients. As a result, conventional p-values would likely overestimate statistical significance. Instead, robustness of the observed relationships was evaluated through multiple complementary approaches, including sensitivity to smoothing, window size, and a moving window correlation analysis, as well as comparison between Pearson and Spearman correlations (see new Table 1, and supplementary Figs. 8 and 9). Pearson and Spearman correlations show very similar temporal patterns, indicating that the observed relationships are robust and not driven by outliers.

Please provide the spectral content of your record in the supplemental information. Any frequencies linked to long-term NAO and/or SSTs as well as external forcing?

Reply: Following the reviewer's suggestion, we performed an exploratory spectral analysis of both the Nb record and the NAO indices (see figure below). The spectra show rather broad maxima at low frequencies (multi-centennial to millennial periods), including enhanced power around ~2,000 and ~5,000 years in the Nb record and around ~4,000 years in the Greenland NAO reconstruction. However, these features are broad rather than sharply defined, occur close to the longest resolvable periods of the records, and can therefore not be confidently attributed to a specific forcing mechanism such as NAO variability, SST variability, or external forcing. We have therefore chosen not to include the spectral analysis also to avoid overinterpretation.*

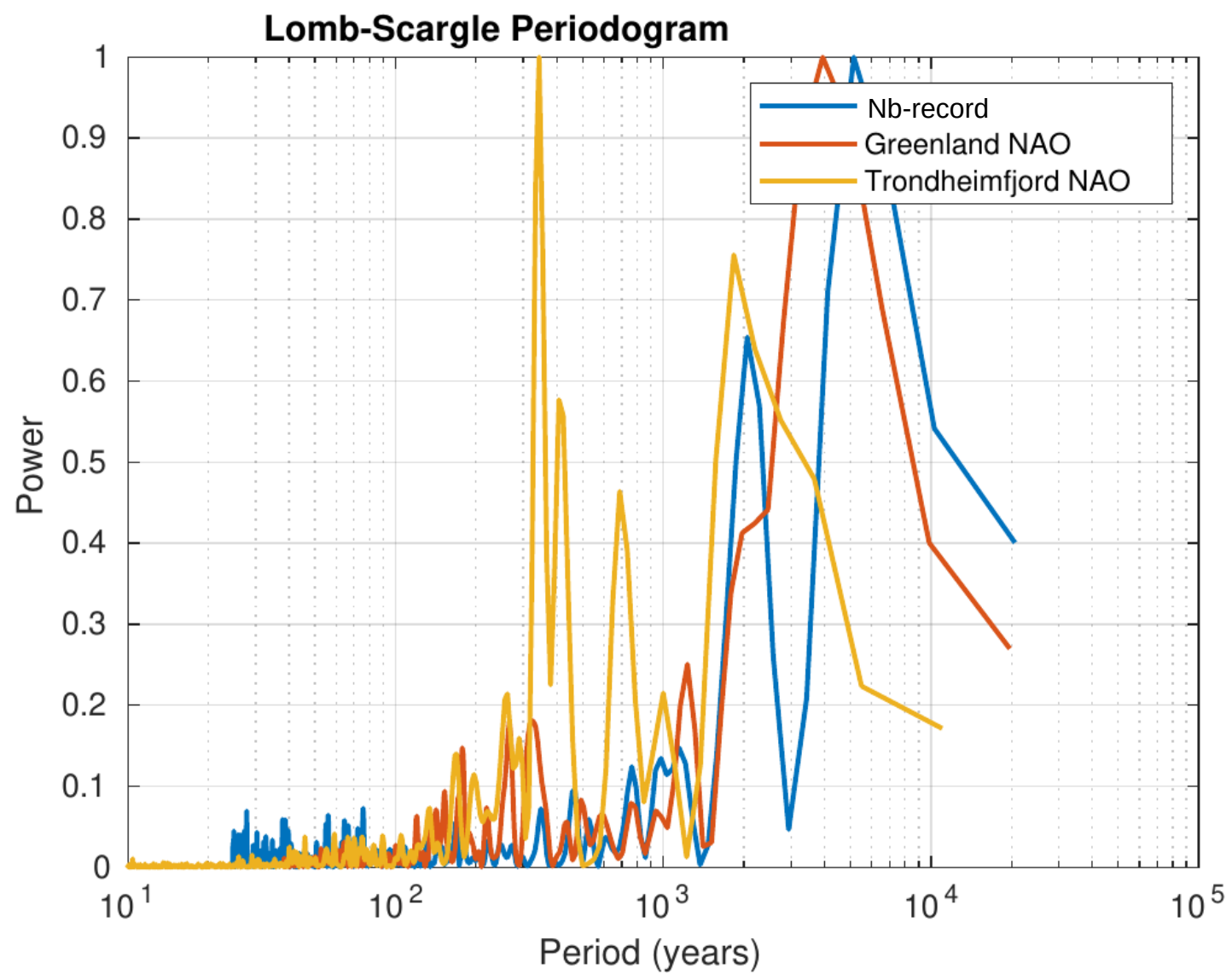

Lines 245-249: please rephrase

Reply: The sentence has been rephrased.

Reviewer #2:

Main comments

With interest have I read this manuscript, which is generally well written and clear. I was triggered to review this paper by the last sentence of the abstract, which states that the Greenland Ice Sheet (GIS) may grow locally due to future changed precipitation patterns.
In essence, the manuscript consists of two components. The first component is the description and analysis of the presented proxy record from southern Greenland. This component is well and clearly written. However, I have to admit that paleorecords are out of my expertise, so opinions of other reviewers on this component should prevail over my opinion. The second component is the interpretation and implications of the results from the proxy records. Again, this part is well written, however, to my opinion the core interpretations of the manuscript are currently not sufficiently proven. These interpretations need either more evidence, or otherwise, must be rewritten in a next version of this manuscript.

The first interpretation is that the proxy record, through the Nobium concentration, demonstrates that local precipitation is, since 5 ky BP, is governed by NAO. This is demonstrated in Figure 5, which is visually rather convincing. However, next to this qualititve match, the authors should provide quantitative evidience of this NAO steering, hence, what is the correlation between the Nobium record and the various NAO index proxies. It might be that some temporal smoothing is needed to get reasonable correlations - I leave that to the authors to figure out and motivate choices on this matter.

Reply: In the revised version of our manuscript, we now provide quantitative evidence for the relationship between the Nb record and the NAO indices shown in Fig. 5. Specifically, we have added "Table 1" presenting Pearson correlation coefficients for our Nb record and each of the NAO reconstruction. In addition, we performed a sensitivity analysis to evaluate the influence of temporal smoothing and window size on the correlations (see Supplementary Figs. 8 and 9). We have added the following sentences to the main manuscript (line 246-254): "Smoothing- and Moving-window correlation analysis of our Nb record with the Greenland and Trondheimsfjord NAO indices reveal consistently positive Pearson correlation coefficients are (Table 1; Supplementary Fig. S8 and S9), reflecting the linkage of atmospheric circulation and Nb content in Narsaq Sound sediments. Correlation coefficients (r) increase systematically with increasing smoothing window, and the relationships strengthen and stabilize with increasing moving window size, indicating that the coupling between the proxies is primarily expressed at centennial

to millennial timescales, while high-frequency variability is dominated by noise and local to regional environmental and climatic processes.”.

The second interpretation in line 279-280, namely: "Collectively, our results demonstrate that southern Greenland hydroclimate and glacier dynamics were strongly modulated by NAO variability" Figure 5 (and additional statistics will) show(s) the link between southern Greenland precipitation and NAO, and Figures 2 and 3 show the link between ice dynamics and climate, hence I do not see any (articulated) demonstration that glacier dynamics is modulated by NAO. As I see the data, as presented now, there is a clear and well known relation between temperature, precipitation and ice sheet dynamics. If the authors think NAO also steers ice dynamics, they should provide more evidence, for example that any/all observed variables presented in Figure 2 correlate with NAO proxies. If this evidence is not there or cannot be demonstrated, this interpretation cannot be hold.

Reply: *We agree that our original wording implied a direct link between NAO variability and ice sheet dynamics, which is not fully supported by the data we present. We have rephrased the abstract (in line 26-29) accordingly: “While the sensitivity to precipitation variability may change under a warmer climate, our results underscore the importance of integrating shifts in NAO-driven moisture delivery to accurately assess the future stability of the Greenland Ice Sheet.” and we also changed the conclusion to “Collectively, our results indicate that NAO variability appears to have strongly modulated southern Greenland hydroclimate and therefore influenced local glaciers and ice sheet dynamics.” (lines 305-307).*

The third and last interpretation is that the proxy data rise the possibility that the projected increase of NAO will allow for regional expansion of the Greenland Ice Sheet. Next that I am not convinced that it is demonstrated that the NAO stir local glacier dynamics, I see a fundamental problem. The projection that the atmosphere will exhibt in the future a preference for a NAO+ state is derived from Earth System Model (ESM) simulations. At the same time the surface mass balance (SMB) and ice sheet projections that project that the GIS will shrink, are based on ESMs too. Hence, if the possibility of the authors is real, then either publications on the future SMB must have overlooked that this region in southern Greenland will have an increasing SMB, or the SMB projections (based on regional climate model (RCM) simulations) all have used ESM realisations in which the NAO does not increase as is anticipated by the authors. By chance, I have such a future SMB projection at hand, and this realization has both a clearly increasing NAO. I've checked the modelled SMB for depicted region with increased SMB in Figure 4-4. In this realization I have at hand, the precipitation over the ice sheet does not significantly increase while melt and runoff increase considerably - hence, also here the Greenland Ice Sheet will retreat. It could be, of course, that the realization that I have at hand is one-off. Still, it is to the authors to demonstrate that either the available SMB projections over Greenland do not have this NAO+ effect which is prominent in the whole ESM ensemble, or that this local SMB increase is there in the RCM projections, but nobody has noticed before. In the end, the conclusion that the GIS might locally grow in the future is bold (and might give rise to the erroneous

interpretation that GIS will florish in a future warmer climate), and hence cannot be based on the interpretation of proxies only, especially when there are ample projections available to evaluate the validity/likelyhood of this claim. Subsequently, if the authors do not, or cannot provide this additional support for the conclusion that this part of the GIS might expand (slightly) in the future, the conclusion on how an increasing NAO influences the sourthern GIS must be reformulated.

<u>Reply:</u> We agree that our proxy-based interpretation does not allow robust projections of future GIS behaviour. As detailed above, we have therefore rephrased the manuscript to frame this aspect more cautiously, emphasizing the mechanistic link between NAO variability and regional hydroclimate rather than making explicit predictions about future ice-sheet change.

While we cannot rule out that precipitation changes may be of secondary importance compared to the primary temperature signal under future warming and certainly do not want to give the impression that the Greenland ice sheet may flourish in such a climate, a significant role of precipitation anomalies under various degrees of warming, including the future, is consistent with our previous work (Born et al., 2019, https://doi.org/10.5194/tc-13-1529-2019). Running BESSI with a range of temperature anomalies resulted in a decreasing SMB over Greenland as a whole (Fig. 13 below) and for all regions (Fig. 14a below). These temperature anomalies were taken from an ESM simulation that covered the years 1850-2100 (SSP5-8.5). When in addition to the T-anomalies, we also imposed the corresponding P-anomalies, the SMB decrease with higher temperatures was markedly offset, in particular in southern Greenland (Fig. 13, 14b). For more details see Born et al., 2019.

REDACTED

REDACTED

<u>Minor comments:</u>

L33: Please use Greenland Ice Sheet, or Greenland Ice Sheet and Ice Caps, or similar, but not this - it is also an one-off.

Reply: *We now use "Greenland Ice Sheet"*

Figure 4: Please specify which time-period is covered by the displayed data, and which NAO definition is used.

Reply: The information which was provide in the method section is now also shown in the figure caption of Fig. 4.

Figure S7: Please display the ice sheet edge in model data. Furthermore, it seems that there is mostly a tundra-runoff response. Why is there very little response in the runoff from the ice sheet?

Reply: The ice sheet edge is now included in supplementary Figure 7. The reason for little runoff from the ice sheet is: it is getting both wetter and colder at the same time. Above a certain elevation—where it's already quite cold—this leads to greater accumulation (supplementary Fig. 7). Below that, there's simply more runoff. It's perhaps important to note that "runoff" here includes all water, meaning rain as well as meltwater.

<u>Reviewer #1</u>

<u>Main comments</u>

I am overall satisfied with the authors` responses. One last comment: Did they authors try to compare their Nb record to reconstructed AMOs (Mann et al. 2009, Wang et al. 2017 & Lapointe et al. 2020)?

<u>Reply:</u> Thank you, we are pleased to hear that.

Regarding the comparison between our Nb record and AMO variability; this is certainly an interesting question, as this would also indirectly indicate a link between NAO and AMO. Following the reviewer's suggestion, we performed a preliminary comparison between the AMO (AMV) reconstruction from Lapointe et al., (2020) and our Nb record (figure below). While some similarities (e.g., in long-term trends) can be seen, the relationship is less clear than for the NAO reconstructions shown in Fig. 5 and was not assessed quantitatively.

Although we believe that a linkage between NAO and AMO is plausible, the relation is certainly complex and may vary through time as e.g. discussed by Seip and Wang 2022 (https://doi.org/10.3390/cli10050063), Börgel et al. 2020 (DOI: 10.1088/1748-9326/aba925) and Sun et al. 2019 (DOI: 10.1007/s00382-018-4201-8*). Therefore, a comparison between these two climate patterns requires a detailed and careful investigation to test the relationship. In the presented study, we show that changes in the elemental composition of marine sediment core GeoB25206-2 are influenced by local temperature and precipitation changes which are linked to large-scale atmospheric circulation patterns. While AMO variability may also affect these relationships, we believe that a detailed investigation of AMO-NAO interactions lies beyond the scope of this study. We therefore cannot draw any further conclusions from this comparison (or include it in the manuscript). However, in the revised version of the manuscript we now note that it may be an interesting topic for future research and to clarify that also other factors (beside NAO) probably affected the sedimentary record and the hydroclimate during the Holocene: line 303-311 " We note that besides changes in NAO, other factors probably affected the sedimentary record and the hydroclimate during the Holocene. For example, sea surface temperature (SST) variability may have influenced regional hydroclimate and could have contributed to the observed trends. However, continuous, high-resolution SST reconstructions from coastal southern Greenland covering the full Holocene needed for testing such associations are currently lacking. Moreover, changes in the Atlantic meridional overturning circulation (AMOC) are unlikely to have been a primary driver of the observed hydroclimatic changes in the time period of interest, as there is currently no evidence for sufficient changes in the AMOC during the past 5 ka [e.g.78].".*

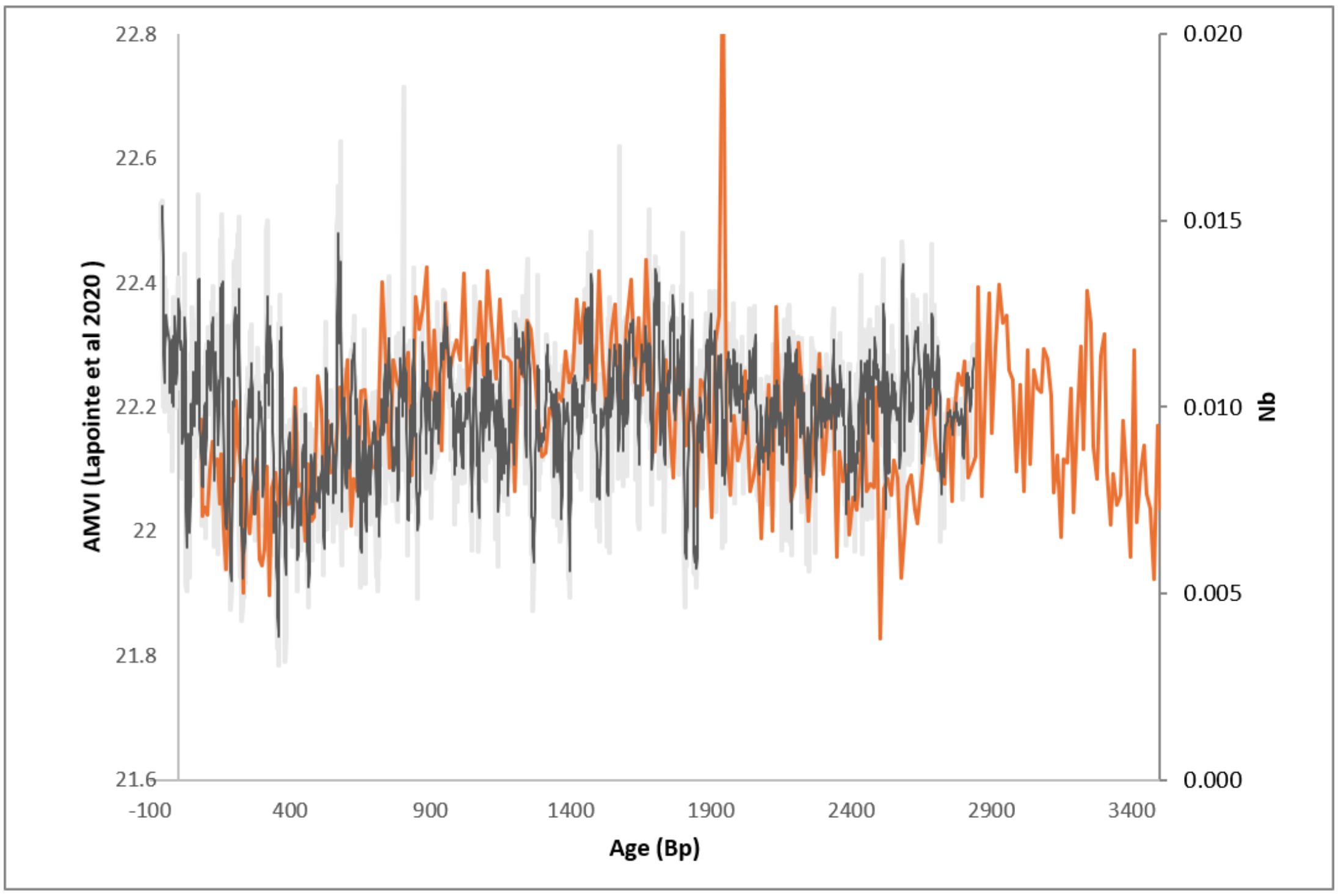

Orange: Nb record (this study), grey: AMV record (Lapointe et al., 2020), black; 5-point running average of the AMV record.

Reviewer #2:

Main comments

In my first review I raised 3 main concerns, and the authors have addressed these. However, I must admit I'm not fully convinced that my concerns are sufficiently covered.

The first concern was the relations shown in Figure 5 were only demonstrated in words, not in numbers. This is sufficiently addressed by the authors. Nonetheless, it would be good if the authors include in the supplementary materials the smoothed version of the records which were used to derive the running correlations shown in Figure S9.

> *Reply: In the revised version of our manuscript the smoothed version of the records used to derive the running correlations shown in Figure S9 are now included in the supplementary table 4.*

The second concern was that Figure 2&3 does not show - as far as I can see - any relation between NAO and ice sheet/glacier dynamics. I asked the authors either to 'articulate' this

relation, that is, describe (and prove) clearly in the manuscript, or refrain of making conclusions. The authors choose to 'soften their claim', thus did neither one of the two, and that is a pity. Even though it is demonstrated that NAO variability modulates the precipitation amounts in this region of Greenland, that does not necessarily imply that glacier dynamics is considerably influenced by this variability. The amplitude or period might be too short to have a measurable impact, after all an ice sheet responds slow. Yes, it may have influenced local glaciers and the ice sheets surface mass balance, but no evidence has been provided that ice sheet dynamics has been altered by NAO variability. So again, prove that that there is a NAO signal in local ice sheet dynamics, or refrain of claiming this.

Reply: *We agree, Figures 2 and 3 do not directly show a relation between NAO and ice sheet/glacier dynamics. Figures 2 and 3 document environmental, hydroclimatic, and glacier-related changes through the Holocene, whereas Figures 4 and 5 provide the evidence linking regional hydroclimate to NAO variability. The influence of NAO on glaciers is thus inferred indirectly through its effect on regional hydroclimate, particularly precipitation, which in turn influences glacier mass balance and glacier behaviour e.g. Mosley-Thompson et al. 2005 (doi:10.1029/2005GL024776); Calder et al. 2008 (https://doi.org/10.1029/2007JD009227); Appenzeller et al. 1998 (DOI: 10.1126/science.282.5388.446); Björk et al. 2017 (https://doi.org/10.1038/s41558-017-0029-1); Buarque and Melia 2018 (https://doi.org/10.5194/cp-14-1707-2018) and Brils et al. 2023 (https://doi.org/10.1029/2023JF007082). We agree that our new data and findings in combination with the published work on NAO impact does not provide a prove, however, we believe it provides enough evidence for our suggestions about the relation between precipitation changes linked to NAO which affects glacial activity and SMB changes. To make this distinction clearer and to place our findings in a broader context, in the revised version of the manuscript we expanded the discussion and included several observational and modelling studies showing that NAO variability influences glacier mass balance, glacier fluctuations, and ice-sheet behaviour across Greenland: Line 268-275 "Our results indicate that NAO variability influenced regional hydroclimate, particularly precipitation, which in turn likely affected local glacier behaviour and ice-sheet mass balance. Even though our data do not demonstrate a direct relationship between NAO variability and glacier or ice-sheet dynamics, several observational and modelling studies show that regional changes in ice volume and glacier fluctuations across Greenland are linked to precipitation variability associated with the NAO [e.g. 70, 71, 72, 73 74]. Consistent with these findings, we suggest that the observed persistent NAO-driven hydroclimate variability in southern Greenland influenced local glacier mass balance and glacier behaviour."*

In addition, we revised the Conclusions to separate the demonstrated relationship between NAO and hydroclimate from the inferred response of glaciers and the ice sheet: Line: 320-322 "Collectively, our results indicate that NAO variability appears to have strongly modulated southern Greenland hydroclimate and changes in hydroclimate likely influenced local glacier behaviour and mass balance." and line 333-338: "Consistent with our findings, investigation of regional changes of Greenland's eastern peripheral glaciers were shown to be related to changes in precipitation associated with the NAO[74]. Based on this relation it has been proposed that they may remain relatively stable under the projected trends towards a more positive NAO in the future [74]. Moreover, the sharp regional contrasts of the SMBs underscore

the need to consider regional climate patterns when extrapolating from local sedimentary and glacier records to whole-ice-sheet behaviour. "

The third concern was the evidence for the conclusion that with future increasing NAO conditions, the ice sheet might locally grow. The authors mention in their rebuttal results of fast and simplified RCM (BESSI), driven by CESM1 data. Although there is indeed some green (increasing SMB) in panel 14b of this figure, there is still, also on the ablation zone in the region of interest, dark red, thus sharply decreasing SMB). So, there is indeed compensation, in these simulations, by increasing precipitation, but as I see it, there is still no offset - and if there is so, the authors could easily demonstrate that from their results by integrating the SMB difference over the drainage basin of the glaciers that are presumed to grow. Besides, that, using a fast and simplified RCM (intended for long (>100 ky) ice sheet simulations) to prove the point is not the optimal model, as there also 'normal' RCM data available for this time scale. Again, I am not aware that any of the RCM projections published (or shared unpublished) give the possibility for an expanding ice sheet in the south.

Reply: *BESSI is not an RCM. It's efficiency comes from the fact that it does not simulate atmosphere dynamics while the mass balance part is rather similar to that of common RCMs. That being said, our main point does not depend on BESSI, but on freely available data from the de-facto standard RCM for Greenland, MAR 3.14. Panels 1 and 2 of figure 4 in our manuscript are solely based on MAR and show, like previous studies, that a positive NAO anomaly leads to an increase in precipitation over southeastern Greenland. The BESSI results in panels 3 and 4 'translate' these anomalies into glaciological variables of relevance, but they naturally also only represent the period covered by MAR, i.e., 1940-2025. While we concede that the phrasing of our original submission may have been ambiguous, we reject the notion that we make claims about a future expansion of the Greenland ice sheet.*
In our previous reply, we included figure 14 of Born et al. (2019) to illustrate the point that also many other studies make, that increases in moisture transport in a warmer climate may very well lead to a compensating effect for glacier mass balance. These earlier results are based on climate change from 1850-2100 (RCP 8.5), so that the linear trend in figure 14 largely represents future climate change. It is to be expected that the positive effect, the "green region" in figure 14b of Born et al. (2019), extends closer to the coast. That is indeed the fact for the new dedicated simulation that we included in the manuscript, with the well-documented caveat that they only cover the observational period as stated above. Additional simulations of future climates are out of scope for the present study that primarily focuses on the novel proxy data and glacier activity of the past.

In conclusion, we believe that we can convincingly show that changes in hydroclimate have an appreciable effect on glacier mass balance in our target region, based on a) our previous work, b) new simulations, and c) our review of the literature. The primary objective of these results is to support the interpretation of the new proxy data, which we find to be consistent. Taken together, we conclude that assessments of past ice sheet stability must take into changes in

hydroclimate into consideration and that a future increase in precipitation in the region, which is to be expected based on CMIP6 simulations, may partially counteract the effect of warming. Our study highlights a potentially important hydroclimatic mechanism influencing the cryosphere of Greenland which will help to further improve climate modelling approaches. It is not our aim to challenge existing future projections; our modelling results are in agreement with several observational and modelling studies showing that regional changes in ice volume and glacier fluctuations across Greenland are linked to precipitation variability associated with the NAO.

Concluding, in my opinion the model and proxy data doesn't provide enough evidence that NAO influence the ice sheet dynamics and may give rise to a local expansion of the ice sheet. If I was the author of this paper, I would refrain thus from writing down both conclusions, even in a "may be"-form.

Reply: In the presented study, we show that changes in the elemental composition of marine sediment core GeoB25206-2 are influenced by local temperature and precipitation changes which are linked to large-scale atmospheric circulation patterns. We believe that our sedimentary data and results combined with our modelling approach and the newly included references form a sufficient base to provide suggestions about the relation between precipitation changes linked to NAO which affects glacial activity and SMB changes. This needs to be considered when investigating Greenland local glacier activity and GrIS behaviour in the past and in the future.

Additionally, I realized that the major local change that could impact the GrIS, if happening, is a significant change in the AMOC. The impact of the cooling ocean south-east of Greenland is a reuced precipitation and a reduced increase of the temperature; and overall might slow down the mass loss of the GrIS. There is, as far as I know, limited published work on it, but existing literature does demonstrate that changes in the ocean temperatures have a distinct impact on the glaciers of Iceland, which is upstream of south-east Greenland. Anyhow, what I wish to convey: the best projections of the upcoming century come from ESMs and RCMs. These models aren't perfect, and past climate reconstructions through proxy data can help to find missing climate processes. In this case, a future changing NAO is (well) represented by the ESMs, and the authors fail to demonstrate that the modelled resulting impact on the GrIS, namely marginal, is incorrect.

Reply: Our study highlights a potentially important hydroclimatic mechanism influencing the cryosphere of Greenland which will help to further improve climate modelling approaches. It is not our aim to challenge existing future projections; our modelling results are in agreement with several observational and modelling studies showing that regional changes in ice volume and glacier fluctuations across Greenland are linked to precipitation variability associated with the NAO.

Lastly, the authors mention never variability in the AMOC, even though this has a major impact on the ice sheet. Yes, there is no indication that during the considered period the AMOC changed significantly, but it would be better if the authors mention that changes in the AMOC is unlikely a cause of the changes in the time period of interest, as there is no

evidence of any significant changes in the AMOC during the past 5 ky (e.g. Pöppelmeier et al, 2023, NatGeo, https://doi.org/10.1038/s41561-023-01140-3).

<u>Reply:</u> We agree that the AMOC may influence the GrIS. However, as the reviewer mentioned, there is currently no evidence for substantial AMOC changes during the period considered here (i.e., the past 5 kyr), making it unlikely that AMOC variability was a primary driver of the hydroclimatic changes observed in our record. To clarify that the AMOC is unlikely to have played a major role for the changes in the time period of interest, we added the suggest reference and the following sentence to the revised manuscript: Line 303-311 " We note that besides changes in NAO, other factors probably affected the sedimentary record and the hydroclimate during the Holocene. For example, sea surface temperature (SST) variability may have influenced regional hydroclimate and could have contributed to the observed trends. However, continuous, high-resolution SST reconstructions from coastal southern Greenland covering the full Holocene needed for testing such associations are currently lacking. Moreover, changes in the Atlantic meridional overturning circulation (AMOC) are unlikely to have been a primary driver of the observed hydroclimatic changes in the time period of interest, as there is currently no evidence for sufficient changes in the AMOC during the past 5 ka [e.g.78]."

<u>Minor comments:</u>

Figure 2:
It might be visually helpful to add dashed colored lines to see quantitative changes better. Like a brown dashed line for 700 (or 750) HU for the sediment density line (just below/trough the minimum values @ 5 ky BP) and a red-brown dashed line at 0 clast/cm3 for the IRD.

> *<u>Reply:</u> We find that the suggested modifications unnecessarily enlarge the figure and does not provide a visual improvement (see figures below). To make it easier to see the quantitative changes, in the revised version of the manuscript we updated Fig 2 by swapping the x and y axis.*

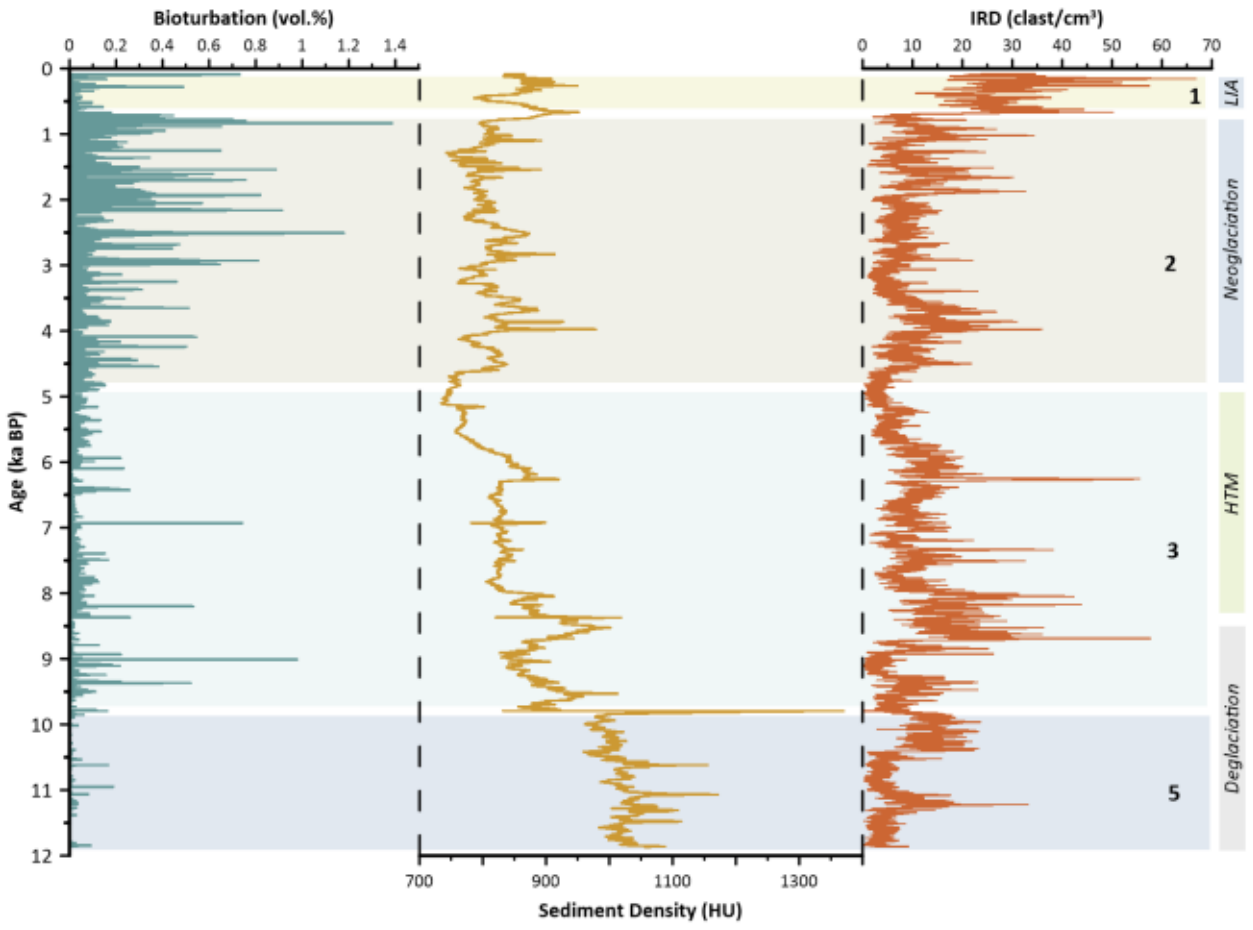

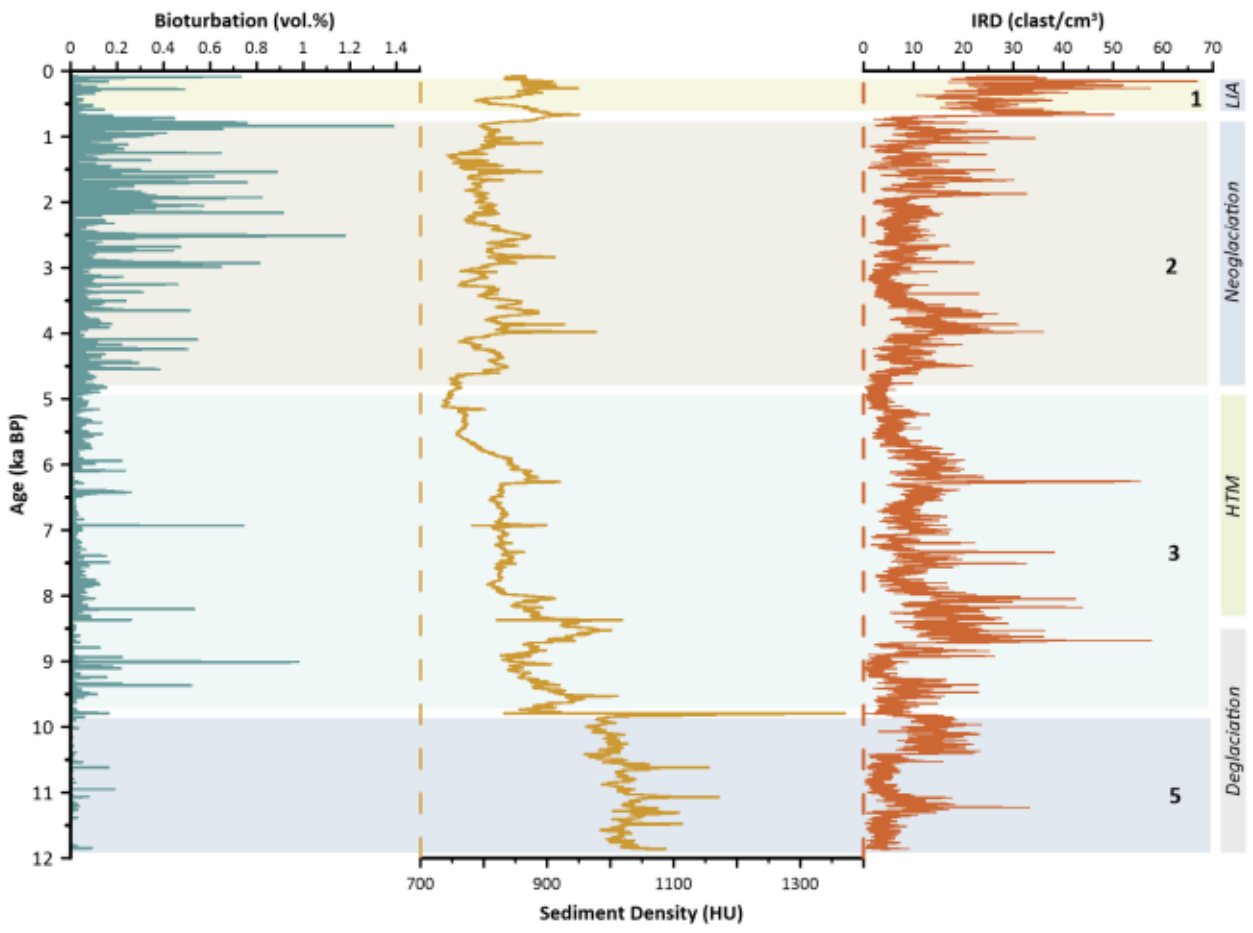

L447: "All data of sediment core GeoB250206-2 supporting the findings of this study are..." Please rewrite to remove the suggestion that the core parts that weren't supporting the findings of this study are not available on PANGAEA

<u>Reply:</u> *We rephrased the sentence accordingly.*

<u>Reviewer #2:</u>

<u>Main comments</u>

3rd review of
Persistent influence of the North Atlantic Oscillation on Late Holocene hydroclimate in southwestern Greenland

The remaining two concern in the 2nd review were:
• Figure 2&3 does not show - as far as I can see - any relation between NAO and ice sheet/glacier dynamics,
• The evidence for the conclusion that with future increasing NAO conditions, the ice sheet might locally grow.
The authors extensively discuss these two remaining questions and have adjusted the manuscript.

Regarding the first concern: There is ample proof, also from the results presented in this manuscript, that North Atlantic Oscillation (NAO) influences the precipitation and subsequently the surface mass balance (SMB). Of course, the SMB and glacier/ice sheet dynamics are closely linked. However, the bottleneck in the argument is the question of whether the variability in SMB is large and persistent enough to cause a measurable change in ice sheet dynamics. As the authors correctly point out, Figures 2 and 3 do not directly show a relation between the NAO and ice sheet/glacier dynamics. The four references in the rebuttal demonstrate the relation between NAO and SMB, but not between NAO and ice sheet dynamics. I have also checked the five additional references in the manuscript. Osman et al. (ref. 70) find that past ice cap changes are linked to NAO changes, but they also conclude that this will not be leading in the future as warming simply eliminate all accumulation zones. Zhao et al., Smith et al., Mitevski et al. (refs 71, 72 and 73) do not address ice sheet changes. Kucera (ref. 74) is a field report and I cannot see (but yes, the report is long) where it proves the link between NAO variability and observable ice sheet changes. Therefore, I still do not see any evidence here or in the literature that NAO variability is strong and persistent enough to cause traceable/relevant changes in the ice sheet dynamics.

> <u>Reply:</u> *We thank the reviewer for identifying the citation mistake. It seems that during preparation of the revised manuscript, our citation manager database became corrupted, resulting in an incorrect reference list. This has now been corrected, and the intended references cited in our previous response (Mosley-Thompson et al., 2005; Calder et al., 2008; Appenzeller et al., 1998; Björk et al., 2017; Buarque and Melia, 2018; Brils et al., 2023) are now included in the manuscript. We have carefully checked the entire reference list and citation order to ensure consistency throughout the revised manuscript.*

Regarding the second concern, let us not forget that the initial manuscript included the following sentence: "Surface mass balance (SMB) calculations under NAO+ conditions (Fig. 4) reveal, in accordance with our late Holocene sedimentary data, that in such a scenario southwestern GIS margins will experience localized growth due to cooler, wetter

conditions, while closely adjacent sectors will exhibit negative SMB implying enhanced ice loss." The second version read: "Surface mass balance (SMB) calculations under NAO+ conditions (Fig. 4) are consistent with our late Holocene sedimentary data, suggesting that southwestern GIS margins may experience localized growth under cooler, wetter conditions, while adjacent sectors could exhibit negative SMB and enhanced ice loss", and a similar statement is made in the lastest draft. In their rebuttal, the authors state: "While we concede that the phrasing of our original submission may have been ambiguous, we reject the notion that we make claims about a future expansion of the Greenland ice sheet." I agree this claim is not made for the Greenland Ice Sheet as a whole, but it is certainly made for the southwestern GIS. As stated above, there is no evidence of NAO-induced changes in glacier dynamics. There may have been some, but then it was too small to be detectable in the proxies you present. Furthermore, the best method currently available to project future (southern) Greenland Ice Sheet dynamics is to use dynamically downscaled SMB by regional climate models and run ice sheet models. I have inquired whether any of the RCM outputs available for ISMIP7 may suggest ice sheet or local glacier growth, but this has been ruled out. This ISMIP7 dataset does include ESM forcings with a NAO+ future, as well as numerous MAR simulations. Therefore, if an increase of the SMB is possible locally for an NAO+ future, it would likely be represented in an ISMIP7 realisation. [Again the absence of such a realisation does not prove that it is impossible; that depends on the mean imbalance of the basins divided by the variability of the SMB across realisations - and I do not have those numbers to hand.] As stated in an earlier review, I am wary of making bold guesses about future behaviour that could be misinterpreted.

To leave this discussion and our review-resubmit loop, I propose rephrasing lines 292-299 (in 661466_2_related_ms_13065618_th9g1x.docx) as follows:
"Our results indicate that NAO variability influenced regional hydroclimate, particularly precipitation. This, in turn, affected the local ice-sheet mass balance, in line with other studies that demonstrate a relationship between NAO and SMBe.g. 70, refs rebuttal. Our proxy data do not provide evidence that NAO variability subsequently caused changes in glacier and ice sheet dynamics, but neither do they exclude the possibility of small changes. So far, late Holocene..."
and it would be great if the term "small" could be quantified, for example as 'such as local advances of less than a kilometre'

And rephrase 358-368 as follows (removing the last two sentences as they are, in my view, repeating previous sentences. I also don't see how 74, an expedition report, proves anything about the future):
... , some model projections suggest that increasing CO_2 concentrations may favour a more positive and less variable NAO state71-73, 79, 80. Surface mass balance (SMB) calculations under NAO+ conditions (Fig. 4) are consistent with our late Holocene sedimentary data. This suggest that the southwestern GIS margins may be more stable in the future, with reduced glacier retreat compared to other areas of Greenland. Further detailed analyses of NAO+ projections could reveal whether increased precipitation could cause localised glacier growth, or whether enhanced ablation by warming remains the leading mass balance process. Moreover, the sharp ...

If the authors are willing to include these or similar reformulations, I have no longer concerns that needs to be addressed. If not, I will leave it to the editor to decide how to proceed.

<u>Reply:</u> *We thank the reviewer for the suggested edits. We appreciate that the remaining concerns relate primarily to the interpretation rather than the proxy reconstruction itself. In response, we have adopted the suggested reformulations throughout the Discussion and Conclusions to better distinguish between the direct evidence provided by our proxy record and the broader implications for glacier and ice-sheet behaviour.*

<u>Minor comments</u>

About Figure 2: See the uploaded adjusted Figure as explanation what I meant in the previous review. If adopted, move then the supporting horizontal lines behind the data and, maybe, the sediment density starting at 750 HU (and add the dashed ochre line then at 750 HU).

<u>Reply:</u> *We thank the reviewer for the suggestion. We explored the proposed modifications; however, after testing several versions (see also our previous response), we found that the additional coloured reference lines did not improve the readability of the figure and instead made it visually more cluttered. We therefore prefer to retain the current presentation.*
